# Non-Metal-Doped Porous Carbon Nitride Nanostructures for Photocatalytic Green Hydrogen Production

**DOI:** 10.3390/ijms232315129

**Published:** 2022-12-01

**Authors:** Qingqing Lu, Ahmed Abdelgawad, Jiaojiao Li, Kamel Eid

**Affiliations:** 1 Engineering & Technology Center of Electrochemistry, School of Chemistry and Chemical Engineering, Qilu University of Technology (Shandong Academy of Sciences), Jinan 250353, China; 2 Gas Processing Center (GPC), College of Engineering, Qatar University, Doha 2713, Qatar

**Keywords:** porous C_3_N_4_, carbon nitride, heteroatom-doped, non-metal-doped, hydrogen evolution reaction, photocatalysts, water slitting, green hydrogen production

## Abstract

Photocatalytic green hydrogen (H_2_) production through water electrolysis is deemed as green, efficient, and renewable fuel or energy carrier due to its great energy density and zero greenhouse emissions. However, developing efficient and low-cost noble-metal-free photocatalysts remains one of the daunting challenges in low-cost H_2_ production. Porous graphitic carbon nitride (gCN) nanostructures have drawn broad multidisciplinary attention as metal-free photocatalysts in the arena of H_2_ production and other environmental remediation. This is due to their impressive catalytic/photocatalytic properties (i.e., high surface area, narrow bandgap, and visible light absorption), unique physicochemical durability, tunable electronic properties, and feasibility to synthesize in high yield from inexpensive and earth-abundant resources. The physicochemical and photocatalytic properties of porous gCNs can be easily optimized via the integration of earth-abundant heteroatoms. Although there are various reviews on porous gCN-based photocatalysts for various applications, to the best of our knowledge, there are no reviews on heteroatom-doped porous gCN nanostructures for the photocatalytic H_2_ evolution reaction (HER). It is essential to provide timely updates in this research area to highlight the research related to fabrication of novel gCNs for large-scale applications and address the current barriers in this field. This review emphasizes a panorama of recent advances in the rational design of heteroatom (i.e., P, O, S, N, and B)-doped porous gCN nanostructures including mono, binary, and ternary dopants for photocatalytic HERs and their optimized parameters. This is in addition to H_2_ energy storage, non-metal configuration, HER fundamental, mechanism, and calculations. This review is expected to inspire a new research entryway to the fabrication of porous gCN-based photocatalysts with ameliorated activity and durability for practical H_2_ production.

## 1. Introduction

The inevitable overuse of fossil fuels (i.e., crude oil, natural gas, and hard coal) generate massive greenhouse gas emissions which are the main contributors to global warming and climate change and increased energy crisis [1,2]. Ceaseless efforts dedicated to defeating this issue have culminated in the conversion of gas to useful chemicals [3,4,5,6] and the development of green energy resources such as biofuel [7] and fuel cells (i.e., methanol [8,9], ethanol [10], glucose [11], oxygen evolution [10], oxygen reduction [12,13]). Hydrogen (H_2_) is one of the most promising green, renewable, and carbon-free fuel or energy carriers, owing to its impressive energy content, earth-abundant resources (i.e., alcohols, methane, and water), and zero greenhouse gas emission [14,15]. H_2_ is produced through various processes such natural gas reformation [16,17], biological (i.e., bacteria and microalgae), biomass, and solar (i.e., photobiological, photoelectrochemical, and solar thermochemical) [18,19,20,21]. Unlike these methods, the green H_2_ evolution reaction (HER) through water electrolysis driven by electrocatalysts (i.e., Pt, Pd, Ru, and Ir), photocatalysts (i.e., metal oxides, metal nitrides, and metal sulfides), and their composites is more promising [22,23,24,25]. Notably, noble metals or Pt-group elements remain the most active catalysts for HERs; however, their earth scarcity and high cost are critical barriers in low-cost HERs [23].

Distinct from noble metals, porous carbon nitride (gCN) nanostructures are physicochemically durable (i.e., thermally stable up to 600 °C, chemically stable in different solvents, and mechanically robust) and, most importantly, are easily synthesized at large scale from cheap and earth-abundant nitrogen-rich organic precursors (i.e., urea, melamine, thiourea, and cyanamide), most of which are produced as byproducts of various industrial processes [26,27,28,29,30,31]. The landmark event of a photocatalytic HER on gCN materials was ignited by the pioneering work conducted by Wang et al. in 2009, which showed the H_2_ production of 7.6 μmol on bare gCNs and increased loaded Pt nanoparticles (15 μmol) via the addition of Pt after 72 h [32]. This study opened new vistas for rational design and utilization of gCNs as photocatalysts for HERs and other applications. Porous gCNs are semiconductors with a bandgap of nearly 2.7 eV and high adsorption of blue-violet light with a wavelength of (<475 nm) that accelerate the migration of photogenerated electrons along with delayed electron–hole recombination [26,27,28,29,30,31]. Moreover, porous gCNs possess high surface area, low density, rich electron density, and massive active sites, which can accelerate the reaction kinetics and maximize the utilization of elements during HER [33,34,35]. The physiochemical and photocatalytic properties of gCNs are easily modulated via the incorporation of metal or non-metal elements to form various heterojunction structures of Z-Scheme, Type-II, and S-Scheme [33,34,35,36,37]. Heteroatoms (i.e., B, S, O, F, and P) are easily integrated into the skeleton structure of gCNs to optimize their electronic structure, bandgap energy, visible-light absorption, and photocatalytic properties [35,38,39]. Also, Heteroatoms are inexpensive, earth-abundant, and easily handled or stored, which makes them feasible or practical applications [40].

**Figure 1:**
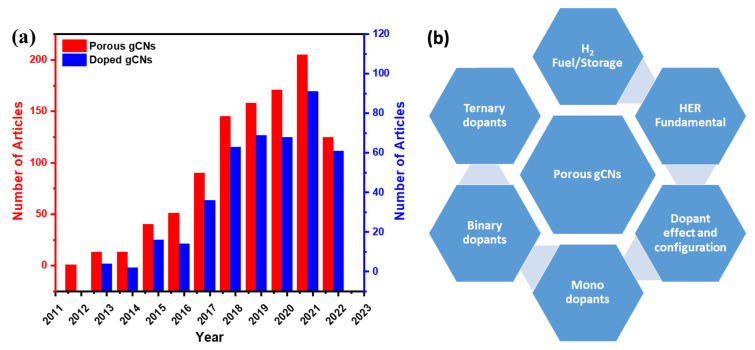
(**a**) The articles related to porous gCNs and doped gCNs published between 2012 and 18 October 2022, obtained from Web of Science data. (**b**) The overall focus of this review.

There have been sensible accumulative efforts in the last decade for the fabrication of porous gCN photocatalysts for H_2_ production, which led to 882 published articles, including 424 articles on doped porous gCN and only 93 articles on heteroatom-doped porous gCNs for H_2_ production (Figure 1a). Therefore, it is essential to provide timely updates in this research area to highlight the research related to fabrication of novel gCNs for large-scale applications and address the current barriers in this field. There are various reviews on porous gCN-based photocatalysts for various applications (Table 1); however, to the best of our knowledge, there are no reviews on heteroatom-doped porous gCN nanostructures for the photocatalytic H_2_ production reaction. 

This review presents the controlled fabrication of porous gCN nanostructures functionalized with mono, binary, and ternary heteroatoms (i.e., P, O, S, N, and B) for photocatalytic H_2_ production. This includes tailoring the physiochemical and photocatalytic merits of porous gCN nanostructures and their photocatalytic H_2_ production activity and durability in addition to dissuasion on the optimization of photocatalytic H_2_ production as a function of catalyst shape, composition, bandgap, and electrolyte type/concentration.

### Novelty and Focus Review

Table 1 shows the focus of the published reviews related to gCN-based nanostructures for H_2_ production compared with our present review. It is obvious that previous reviews focused on gCN fabrication strategies for photocatalytic H_2_ production or other applications. This is in addition to the formation of heterojunctions structures (Z-scheme, S-scheme, and Type-II scheme) via coupling with other semiconductors (i.e., metal oxides and metal sulfides), carbon materials (i.e., graphene, metal-organic framework, and graphdiyne), and metals/non-metals elements (i.e., Pt, Ag, Ni, Mo, F, and B) for photocatalytic H_2_ production and other applications as well. In this review, we collected all the articles related to heteroatom-doped porous gCN nanostructures for H_2_ production from the Web of Science, Scopus, and Scifinder databases without bias. Distinct from previous reviews, the main foci of this review are summarized in Table 1, which includes (1) the fundamentals of water electrolysis; (2) the rational design of porous gCN nanostructures functionalized with mono, binary, and ternary heteroatoms (i.e., P, O, S, N, and B) and exploitation of their unique photocatalytic H_2_ production properties; (3) H_2_ energy and storage; (4) HER fundamental and calculation; (5) discussion on the optimization of the photocatalytic H_2_ production and (6) current challenges facing and future remarks on directing the synthesis of porous gCNs for practical applications (Figure 1b).

## 2. H_2_ as Fuel Strategy

The demand for H_2_ increased by nearly three times in the last 3 decades and is expected to continue to rise to reach the net carbon-free emissions target by 2050. The earth resources for H_2_ are plentiful, including H_2_O and hydrocarbons (i.e., CH_4_), and it is easily extracted from fossil fuels (i.e., crude oil, natural gas, and hard coals), biomass, and water [18,19,20,21]. Currently, natural gas is the main source of H_2_ production (~95%), which not only consumes almost 6% of global natural gas and 2% for production of 70 million tons annually, but also generates a huge amount of CO_2_ emissions [44,45]. Mainly, H_2_ production—from crude oil or hydrocarbons—allows complete formation from carbon to CO_2_, while considering the energy for combustion each 1 million SCF of H_2_ produces 19.3 metric tons of CO_2_.

**Figure 2:**
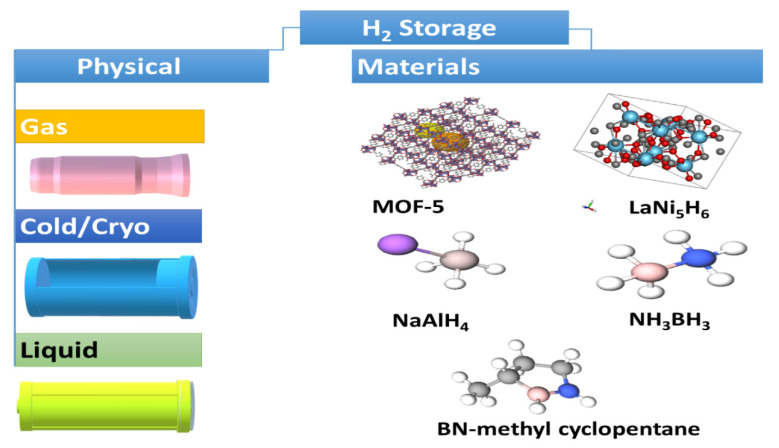
The main H_2_ storage methods.

H_2_ fuel is green and effective, with outstanding energy compared to traditional sources. H_2_ has the greatest energy per mass of any other fuel. For example, 1 kg H_2_ produces 120 MJ of energy, which is equivalent to 1 gallon of gasoline (44 mJ/kg) and provides 9.1 kg of CO_2_ after combustion [14,15]. However, H_2_, due to its inferior ambient temperature density, has a low energy per unit volume. Even in liquid form, H_2_ has an energy density of 8 MJ/L compared to gasoline (32 MJ/L) [14,15]. Thus, H_2_ requires the rational design of an efficient storage system with potential for greater energy density. H_2_ is stored physically as either gas in high-pressure tanks at 350–700 bar or as a liquid that needs cryogenic temperatures owing to the negative boiling point of H_2_ (−252.8 °C) at one-atmosphere pressure [18,46,47,48]. H_2_ can also be stored via the adsorption on the surfaces of adsorbents (i.e., MOF, liquid organic, interstitial hydride, complex hydride, and chemical hydrogen) (Figure 2) [18,46,47,48]. LiBH_4_-based materials are one of the promising materials for hydrogen storage/release [49,50,51,52,53,54]. However, the thermodynamics and sluggish kinetics of hydrogenation/dehydrogenation is a major obstacle in hydrogen storage/release. Research efforts to improve the hydrogenation/dehydrogenation of this material have utilized multiple approaches. Notably, nanoengineering through techniques like ball milling and confinement in a nanoporous scaffold can reduce grain size and particle size as well as increase the number of defects to allow better diffusion of H_2_ and thereby enhanced kinetics of H_2_ adsorption/desorption. Another strategy to improve the kinetics of H_2_ adsorption/desorption is to dope LiBH_4_ with metals such as Ti, metal oxides such as SiO_2_ and TiO_2_, and halides such as TiCl_3_. However, the effect of doping is almost negligible on hydrogen re-absorption [50]. Moreover, tuning the thermodynamics of LiBH_4_ can allow for better hydrogen adsorption or desorption [49,53]. This can be achieved through partial substitution of the metal cation with metals of a lower electronegativity or the BH_4_ anion by elements such as F to yield more thermodynamically favorable adsorption/desorption. Notably, hybrid materials made of Mg(BH_4_), LiBH_4_, and MgH_2_ using a novel fabrication technique has shown to be promising for hydrogen storage/release: 5 wt% in solid state at temperatures <265 °C [49,50,51,52,53,54,55,56]. The hybrid mix is fabricated using room temperature ball milling of MgH_2_ with in situ aerosol spraying of LiBH_4_. In addition to experimental efforts, machine learning was employed to predict the hydrogen release ability of LiBH_4_ materials and the rank of major variables including sample preparation, mixing conditions, and operational variables [49]. In spite of research efforts to develop and optimize materials for hydrogen storage, the capacity for hydrogen storage is currently very limited and requires high temperatures, which makes it energetically unfavorable. However, further investigations are still needed to develop new storage methods for tanks for more effect delivery and to avoid current challenges. H_2_ density shortage remains a daunting challenge for transportation applications, as it needs huge volume systems in gaseous form, so fuel-cell-powered vehicles contain compressed gas onboard storage with large-volume and high-pressure composite vessels that can withstand driving for more than 300 miles and can accommodate large H_2_ storage capacity (5–13 kg) onboard [14,15,18,46,47,48].

### Solar-Driven H_2_ Production

The utilization of solar energy in green H_2_ production could be carried out through two main approaches including water electrolysis using solar-generated electricity and direct solar water splitting [18,19,20,21]. However, the solar-driven electricity “photovoltaic-electrolysis-fuel cell’’ should soon be available to make solar-driven H_2_ production a feasible process, which is not possible in remote areas or during seasonal variations.

Photovoltaic electrolysis-driven H_2_ production, especially for automobile applications, is not feasible supposing the fueling station needs nearly 1000 kg H_2_/day and considering that the minimal electrical energy required for production of 1kg H_2_ is 51 kWh (utilizing an electrolyzer efficiency of 65%) [33,34,35,36,37,38,39,40]. Thereby, 1000 kg H_2_/day needs 51,000 kWh/day of electricity that requires operation of 10,200 kWp or 10.2 megawatts of PV power. Notably, 1 kWp needs about 10 m^2^ area for PV at 10% efficiency.

Direct solar H_2_O splitting entails the direct utilization of solar energy in the production of H_2_ from water without going through intermediate electrolysis, which includes the following concepts [33,34,35,36,37,38,39,40]:

- Photoelectrochemical H_2_O splitting driven by quantum dots or semiconductor (i.e., electrodes using a photoelectrochemical cell) to convert light energy into H_2_ chemical energy. Photoelectrochemical systems could be based on semiconductors or dyes and using dissolved metal complexes.
- The photobiological process includes the production of H_2_ from biological systems (i.e., algae and bacteria using sunlight driven by the initial absorption of light by the pigments in algae while the enzymes in the cell act as catalysts to promote H_2_ or O_2_ production). Both photoelectrochemical and photobiological approaches should be improved significantly to meet large-scale applications because current solar-to-H_2_ systems’ efficiencies are less than 1%.
- Thermochemical cycles for generating high temperature from solar light to produce H_2_, which can achieve efficiencies higher than 40%. However, it needs a concentrated solar receiver/reactors able to generate a high temperature of nearly 800 °C.

According to the US department of energy, photocatalytic water electrolysis is the main pathway for H_2_ production to reach the H_2_ energy earth-shot aim of decreasing the cost of H_2_ by 80% to nearly 1 USD/kg along with zero greenhouse gas emissions by 2031 [57,58,59]. In spite of the great progress made in photocatalytic HERs, the current efficiency is not up to a level that meets practical requirements. In particular, considering daily sunlight illumination (AM 1.5 G) of 7.6 h (assigned to 240 W/m^2^), to reach a hydrogen price of 3.5 USD/kg, it is estimated that a HER system with an STH of (10%), lifetime of (10 years), decreasing rate of 4%/year, and subsequent acceptable cost of 102 USD/m^2^ is required. Therefore, further efforts including both experimental and theoretical studies along with fundamental investigations are needed to develop efficient and low-cost photocatalysts for large-scale photocatalytic HER processes [33,34,35,36,37,38,39,40].

## 3. Fundamentals of HER

### 3.1. Photocatalytic HER Mechanism 

The photocatalytic water-splitting reaction on gCN to produce H_2_ comprises three consequent main steps as follows [33,34,35,36,37,38,39,40].

The initial absorption of photons when photon energy is ≥ than the bandgap of gCN to generate electron–hole pairs (e^−^/h^+^) via the excitation of electrons from the valence band (VB) to the conduction band (CB) while the holes are left in the VB:

(I) The isolation of the photoexcited carriers into free carriers followed by migration to the active sites of gCN.
(II) The initiation of a reduction reaction comprising these charges to produce H_2_ on the surface of gCN with the assistance of e^−^ in the CB. The HER reaction in different electrolytes is shown in Equations (1) and (2)

In an aqueous solution of acidic electrolyte

2H^+^ + 2e^−^ → H_2_ (1)

In an aqueous solution of alkaline electrolyte

2H_2_O + 2e^−^ → H_2_
*+* 2OH^−^ (2)

The overall solar HER efficiency (*η*_total_) is estimated via the kinetics of the above steps together according to Equation (3):

*η*_total_ = *η*_absorption_ × * η*_separation_ × *η*_reaction_ (3)

where *η*_absorption_ is the light absorption efficiency that is the fraction of generated e^−^/h^+^ pairs excited by the incident photon flux, *η*_separation_ is the charge separation efficiency that is the fraction of photogenerated charge carriers that isolate and migrate to the solid–liquid interface, and *η*_reaction_ is the reaction efficiency of the gCN surface; that is, the efficiency of the surface reaction involving charge carriers at the solid–liquid interface.

**Figure 3:**
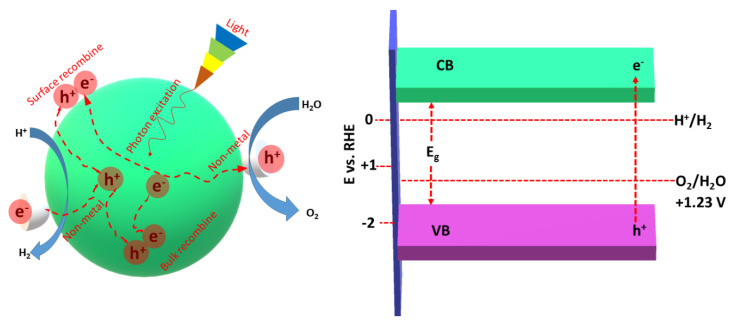
The role of non-metal dopants and the photocatalytic HER mechanism using non-metal doped porous gCN (**left**) and the band gap of gCNs relative to the required energy barrier for water splitting (**right**). This figure was designed based on the data in Ref [61].

Notably, the lowest band edge of the CB should be lower than the redox potential of H^+^/H_2_ (0 V vs. RHE), and the highest band edge of the VB should be greater than the chemical redox potential of O_2_/H_2_O (Figure 3) [60,61]. Non-metal atoms provide more active sites and reaction centers on the surface of gCN, allowing delay recombination of e^−^/h^+^ [60,61]. Owing to the ultrafast rate of e^−^/h^+^ (~ps to ms), they should be promptly captured by the non-metal atoms for promoting the separation and migration of photogenerated charges along with participation in the HER.

### 3.2. Electrocatalytic HER Mechanism 

Table 2 summarizes the electrocatalytic HER mechanisms in aqueous solutions of both acidic and alkaline electrolytes. In an aqueous solution of acidic electrolyte, two or three reaction pathways can take place (Equations (4)–(6)) and the HER ensues on the active site of the gCN electrocatalyst (M*):

(I) A Volmer reaction step that includes a discharge step to allow reduction of protons on the M* and subsequent proton adsorption on M* of gCNs to form gCN-M*H_ads_ (Equation (4));
(II) A Heyrovsky reaction step that involves electrochemical desorption to desorb H_2_ from the M* via the proton/electron transfer and regenerate of M* Equation (5);
(III) A Tafel reaction step that includes the coupling of two adsorbed protons to release H_2_ and the regeneration of M* (Equation (6)).

The HER mechanism in an aqueous solution of alkaline electrolyte (Equations (7)–(9)) includes Volmer–Heyrovsky or Volmer–Tafel reaction steps but with the initial dissociation of H_2_O on the M*, due to inferior or low proton concentration in the alkaline electrolyte.

Therefore, the additional energy needed for dissociating H_2_O in alkaline electrolyte is an additional energy barrier of the HER, consequently affecting the overall reaction rate and kinetics. Thus, the HER kinetics in alkaline electrolytes is extremely sluggish relative to acidic electrolytes, owing to more abundant proton donor presence (i.e., H_3_O^+^) in acidic electrolytes than (i.e., H_2_O) in alkaline electrolytes. However, noble metals can accelerate the HER rate and kinetics in different electrolytes over broad pH ranges, while few noble-metal-free catalysts can do this. Therefore, it remains a grand challenge to develop electrocatalysts or photocatalysts for HERs over different pH ranges (i.e., acidic, alkaline, and neutral).

### 3.3. HER Measurements and Calculations 

The HER process is conducted using various cyclic voltammograms (CVs), impedance spectroscopy (EIS), linear sweep voltammograms (LSVs), chronoamperometry (I-T), Tafel plots, and electrochemical impedance tests in the presence and absence of light. The potential window for a HER in a CV mainly depends on the electrolytes, including (−0.3 to +1 V in the acidic electrolyte) and (+0.3 to −1 V in the alkaline electrolyte); meanwhile, in an LSV the potential direction must be negative (0 to −1 V or 1.5 V)—regardless of the type of electrolyte—to allow for a reduction reaction. The I-T time ranges from a few minutes to several hours based on the catalyst’s durability in the electrolyte solution; however, the long-term durability testing takes several weeks. The activity of the gCN-based photocatalysts is determined using the following calculations, summarized in Table 3.

The quantum efficiency (QE) is calculated using Equation (10) by dividing the number of reacted electrons by the number of the incident photons.

The current density (*J*) is calculated via the dividing of measured current (I) on the geometric surface area of the working electrode (A) Equation (11). The catalysts should be able to produce an excellent *J* value under low applied potential (V). 

The overpotential (*η*) is calculated by Equation (12). *E* is the measured electrode’s potential to deliver a current density of 10 mA/cm^2^ or more and *E^o^* is the standard potential for water splitting (−1.23 V for OER and 0 V for HER). The catalysts should possess a lower *η* to allow fast HER kinetics. 

The turnover frequency (TOF) of each active site is calculated based on *J* and *η* by Equation (13), where 2 is the number of moles of electrons consumed in the evolution of one mole of H_2_ from H_2_O; *F* is the Faradic constant (96,485 C/mol), and *m* is the number of active sites (mol). 

The active sites (m) are extracted from the linear relationship between the reduction current densities as a function of sweeping rates, which show a linear relationship. The slope could be obtained from the graph to be used in Equation (14), where *n* is the number of electrons transferred; *Γ*0 is the surface concentration of the active sites (mol/cm^2^); *R* is the ideal gas constant and *T* is the absolute temperature.

The TOF can also be calculated using Equation (15), where NA is the Avogadro number, F is the Faraday constant, n is the number of electrons transferred (two for HER and four for OER) to generate one molecule of H_2_ or O_2_, and *Γ* is exact number of active sites catalyzing the reaction per square meter. Therefore, catalysts with abundant active sites should provide higher TOF. 

Energy efficiency (*E*_efficiency_) is calculated by Equation (16), where *E*_eq_ is the equilibrium potential; the energetically efficient catalyst should allow HER at a lower *η* and with a high E_Faradic_.

The quantum yield (QY) is calculated from Equation (17), where *n_x_* is the moles of the product; *N_A_* is Avogadro’s constant (6.022 × 1023 mol^−1^); *h* is Planck’s constant (6.626 × 10^−34^ J.s); *c* is the speed of light (3 × 108 m.s^−1^); *t_ill_* is the light illumination time; *I* is the incident intensity (W/cm^2^) and *λ* is the wavelength of light (nm); and *A* is the irradiated area of the cell (cm^2^).

Non-metal-doped gCNs give the CV features of rectangular shape assigned to high capacitance effect, so the electrochemical active surface area (ECSA) is calculated using the double capacitance (C_DL_) via measuring the CV at different scan rates because of N_2_-saturated aqueous electrolytes (Equation (18)), where *C*_s_ is the specific capacitance of a flat surface with 1 cm^2^ (0.015–0.110 mF/cm^2^ in H_2_SO_4_), (0.022–0.130 mF/cm^2^ in NaOH and KOH solutions) depending on the electrolyte concentration [62]. 

Then the double-layer capacitance (*C*dl) is determined via plotting the Δj = (ja-jc) at 0.1 V as a function of sweeping rate using Equation (19). The Cs is assumed as 20.9 μF/cm^2^ for a flat electrode with 1 cm^2^ of real surface area [63]. Higher ECSA is an indicator for the greater active sites.

The incident photon to current conversion efficiencies (IPCEs) are calculated using Equation (20), where λ is the wavelength of the incident light, *J* is the photocurrent density, and *I_o_* is the incident light intensity. Thus, photocatalysts with lower bandgap energy can enhance the viable light absorption and provide higher IPCEs.

The Tafel slope is obtained from Tafel plots via plotting the *η* vs. log *J*. A greater Tafel slope is an indication of quick HER kinetics and high activity.

The EIS fitting and Voigt electrical equivalent circuit are usually used for fitting the EIS data to obtain electrolyte resistance (*R*_s_), charge transfer resistance (*R*_ct_), and constant phase elements (CPE). The photocatalysts should reveal lower *R*_s_ and *R*_ct_ along with higher CPE, made evident by better electrolyte–electrode interaction and quick charge mobility. 

## 4. Role of Non-Metal Dopants 

**Figure 4:**
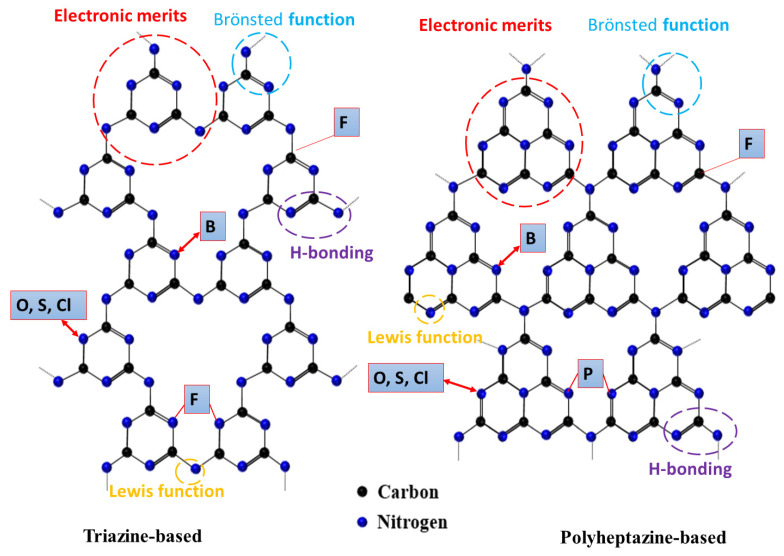
The molecular structures of gCNs and their merits and accommodation sites for non-metal dopants into gCNs based on data from Refs. [28,38,64].

Non-metal dopants can modulate bandgap energy, augment light absorption, facilitate separation of e^−^/h^+^ pairs, attract electrons, and enhance the separation and migration of gCN charge carriers (Figure 4). Moreover, non-metal dopants provide active sites, serve as electron sinks, generate active sites for proton reduction, and improve durability. The minimum theoretical bandgap required to drive the overall water-splitting reaction is 1.23 eV, which corresponds to a wavelength of approximately 1000 nm, so an additional overpotential is usually needed to induce and tune the electron migration process during the HER [60,61]. The reductive decomposition potentials and CBM should be more negative than the H^+^/H_2_ potential to allow thermodynamic durability of gCN-based photocatalysts during the HER [60,61]. 

### 4.1. Integration of Heteroatoms

The chemical structure of gCNs is still questionable; however, the most recognized structures are triazine or polyheptazine, which possess various unique properties (Figure 4). The heteroatom elements are integrated via abundant active sites. Figure 4 shows the possible substitution sites of heteroatoms (i.e., B, O, S, P, Cl, Br, I, and F) in gCNs, including the substitution of N atom with (I, S, and O), coordination of P with two N-atoms and replacement of C with B or coordination with F (C-F) (Figure 4) [60,61]. Heteroatom dopants, with their great inbuilt ionization energies and electro-negativity, can form covalent bonds by gaining electrons when reacting with other compounds. Meanwhile, the physicochemical properties (i.e., size, electronegativity, and chemical state) and location of non-metal dopants can tune the catalytic and photocatalytic properties of gCN.

### 4.2. Non-Metal-Doping Configuration and Effects

Unlike noble metals (i.e., Pt, Pd, Au, and Ru) and transition metals (i.e., Co, Cu, and Ni), non-metal dopants are earth-abundant and inexpensive, enhancing electrical conductivity, hydrophilicity, and active sites [60,61,65]. This is due to electron-donating or electron-withdrawing characteristics that tailor the electronic, catalytic, and photocatalytic properties of gCNs. Non-metal atoms can act as trapping sites facilitating the generation of electron–hole pairs, delaying their recombination, and enhancing light absorption under light illumination [60,61]. Table 4 summarizes the main advantages and disadvantageous of heteroatom-doped porous gCN. 

A N atom, with its size (~155 pm) being close to a carbon atom (~170 pm) but with a larger electronegativity (~3.04) than carbon (2.55) can withdraw electrons, generate abundant active sites, and boost the electronic and ionic conductivity of carbon [6,38,60,61]. Meanwhile, the N atom creates electron-deficiency on its adjacent positive C atom with its negative charge, allowing for a dipole N^−^-C^+^ bond, which makes gCN more feasible for electrophilic and nucleophilic attack in addition to accelerating charge mobility and facilitating the dissociation of H_2_O molecules during a HER [64,66,67,68,69,70]. N-doping into gCN can result in N atom pyridinic, pyrrolic/pyridonic, quaternary/graphitic, and pyridine-N-oxide that show binding energy at 398.5, 400.1, 401.1, and 403.2 eV, respectively, as determined by X-ray photoelectron spectroscopy (XPS) [71]. These N atom species are highly active sites for CO_2_RR and other catalytic applications, which induce Lewis basicity on the C atom and act as active sites during HERs.

The boron (B) atom, with a smaller size of (85 pm) and lower electronegativity (2.04) than C, is inserted into the skeleton structure, as it replaces C in the gCN without affecting its planar structure [35,38,39,40]. B, with its electron-deficiency that accounts for boron being a strong Lewis acid that can easily accept protons, decreases the Fermi level into valance, and promotes charge polarization of gCNs. Moreover, B can decrease the Fermi level into valance, generate multiple defects, enhance visible light absorption, modulate bandgap energy, and enhance the reactivity of gCN [35,38,39,40]. According to the XPS, there are two main in-plane binding structures of B in gCN, including graphitic B at 200.5 eV and B-substituted C atoms in the hexagonal triazine or polyheptazine rings of co-conjugated gCN at 198.5 eV [72]. Interestingly, B can stabilize the negatively polarized oxygenated atoms during the HER, consequently enhancing H_2_O molecule chemisorption from the electrolyte during the HER process. 

Sulfur (S), with a larger size (180 pm) and higher electronegativity (2.58) than carbon, promotes the electrical conductivity and spin density, edge strain, and charge delocalization of gCN via the substitution of the N atom with S in gCN [35,38,39,40]. Thus, the C-S bond in triazine or polyheptazine at the edges of gCN is easily determined by the XPS at a binding energy of 163.7 eV. 

Phosphorus (P), with lower electronegativity (2.19) and large size (195 pm) than C, creates positive charges on P dopant and negative charges on positively charged C. This leads to the formation of (P^+^-C^−^) via the co-coordination of P with two C atoms in triazine or polyheptazine of gCN, which can promote charge mobility and provide plentiful active sites during a HER [35,38,39,40]. A C-P bond is detected at a binding energy of 132.5 eV. 

The oxygen (O) atom has a lower atom size (152 pm) than C but a higher electronegativity (3.44 pm), which increases the positive charge on its neighboring C atom in the form of (C^+^-O^−^) after substitution of N with O in gCNs [35,38,39,40]. This allows more electron donation to generate additional active sites, boosting the electronic and ionic conductivity of gCN. Additionally, the O atom alters the intrinsic electronic structure of gCN, which is important for controlling the binding energies of reactants and intermediates during HER, due to oxidant functional groups (i.e., C-O, C=O, and C-OH), which can induce in situ formation of active oxygen species (i.e., -OH, O_2_) needed for activation and dissociation of H_2_O_2_. The XPS can detect C-O bonds at 532 eV and C=O 533 eV. However, the exact location of heteroatoms in the skeleton structure of gCNs and their effect is still ambiguous. Additional theoretical and experimental studies are needed, along with in situ characterization tools, to determine their position and effects during catalytic and photocatalytic reactions.

## 5. Heteroatom-Doped Porous Carbon Nitride 

Nowadays, various types of carbon nitride nanostructures, including nanosheets, nanotubes, and nanoflowers, have been designed as highly efficient photocatalysts for HERs [65,73,74,75,76]. Heteroatom-doped porous carbon nitride, which combines the advantages of both heteroatom dopants and the porous structure of carbon nitride, can further extend light adsorption, increase active sites, and facilitate charge transfer and separation, thus enhancing photocatalytic performance [60,77,78,79]. There are various approaches to doping of gCNs with various heteroatoms. Table 5 shows the advantages and disadvantages of the main methods for the preparation of doped gCNs. 

### 5.1. Mono Heteroatom Doped Porous Carbon Nitride

Table 6 summarizes diverse mono-heteroatoms including phosphorus [76,89,90,91,92,93,94,95,96,97,98,99,100,101,102,103], sulfur [104,105,106,107,108,109,110,111,112,113,114], boron [115,116,117,118,119,120,121], oxygen [122,123,124,125,126,127,128,129,130,131,132,133,134], carbon [135,136,137,138,139], nitrogen [140,141,142], and halogens [143,144,145,146] doped porous carbon nitride catalysts, and their photocatalytic hydrogen production performance. Moreover, the effect of morphology, preparation methods, and light source on the H_2_ production rate and stability on mono-heteroatom-doped porous gCNs is also summarized in Table 6.

#### 5.1.1. Phosphorus Doping

**Figure 5:**
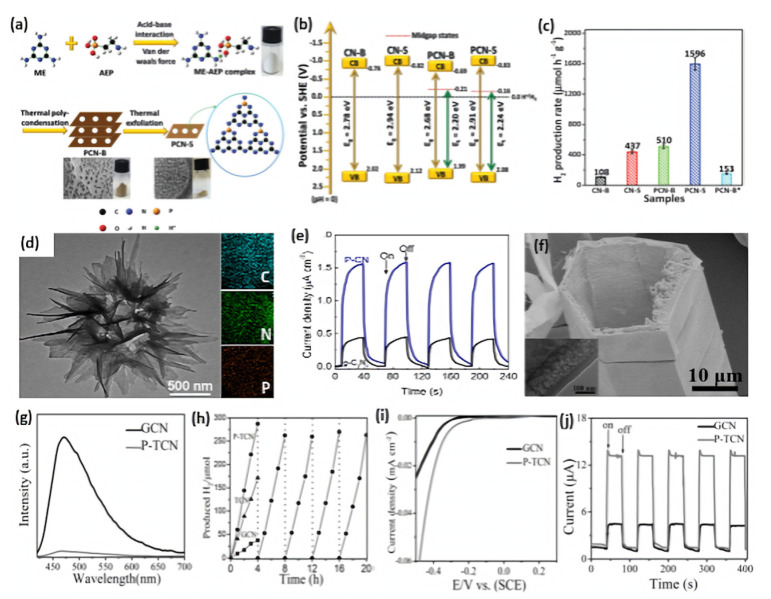
(**a**) Schematic illustration of the synthesis process of porous PCN-S. (**b**) Electronic band structures of CN-B, CN-S, PCN-B, and PCN-S. (**c**) The photocatalytic activity comparison of CN-B, CN-S, PCN-B, PCN-S, PCN-B*; adapted with permission from Ref. [89]. 2015, Royal Society of Chemistry. (**d**) Transmission electron microscope (TEM) image and EDS mapping of P-CN nanoflowers, beside its photocurrent–time curves relative to bulk g-C_3_N_4_ flakes (**e**); adapted with permission from Ref. [76]. 2015, American Chemical Society. (**f**) SEM and TEM images of P-TCN. (**g**) PL spectra of GCN and P-TCN. (**h**) Time course of H_2_ production for GCN, TCN, and P-TCN. (**i**) Polarization curves of GCN and P-TCN. (**j**) Photocurrent–time curves for GCN and P-TCN; adapted with permission from Ref. [91]. 2016, John Wiley & Sons, Inc.

Qiao’s group reported the synthesis of porous P-doped g-C_3_N_4_ nanosheets (PCN-S) by using melamine (ME) and 2-aminomethyl phosphonic acid (AEP) as g-C_3_N_4_ precursor and P source, respectively [89]. As shown in Figure 5a, ME and AEP are first tightly coupled via acid–base interaction and van Der Waals’ force to form a ME-AEP complex. After evaporation and thermal polycondensation, bulk P-doped g-C_3_N_4_ (PCN-B) are synthesized. PCN-S were obtained by thermal exfoliation of PCN-B; thus, numerous macropores generated by AEP decomposition can be exposed. As a reference, bulk P-doped g-C_3_N_4_*(PCN-B*) was prepared by using (NH_4_)_2_HPO_4_ as a P source. Bulk g-C_3_N_4_ (CN-B) was prepared under identical conditions to that of PCN-B except for the addition of AEP. g-C_3_N_4_ nanosheets (CN-S) were prepared by thermal exfoliation of CN-B under identical conditions to that of PCN-S. Based on XPS and calculation results, all the doped P atoms replace the more energy-favorable C site to form a P-N bond in PCN-S. Moreover, unlike the conventional band gap narrowing, they found that P doping can induce the appearance of an empty midgap state in PCN-B and PCN-S (Figure 5b), thus extending the visible light-harvesting ability of the photocatalytic HER. As confirmed by steady-state and time-resolved photoluminescence spectroscopy and electrochemical impedance spectra measurements, PCN-S exhibits the highest separation and transfer efficiency of photo-excited electron–hole pairs. Among these photocatalysts, PCN-S possesses excellent H_2_ production activity of 1596 μmol h^−1^g^−1^, which is 14.8, 10.4, 3.7, and 3.1 times higher than that of CN-B (108 μmol h^−1^g^−1^), PCN-B* (153 μmol h^−1^g^−1^), CN-S (437 μmol h^−1^g^−1^), and PCN-B (510 μmol h^−1^g^−1^), respectively (Figure 5c). Thus, the H_2_ production activity of PCN-S (1596 μmol h^−1^ g^−1^) and apparent quantum efficiency (3.56%) are among the highest-reported metal-free free g-C_3_N_4_ photocatalysts. This is due to the porous morphology and high surface area of PCN-S (122.6 m^2^/g) that was 1.4, 14.7, and 21.8 times of CN-S (84.2 m^2^/g), PCN-B (8.3 m^2^/g), and CN-B (5.6 m^2^/g), respectively. Therefore, not only does a porous nanostructure enhance the surface area, provide more active sites, and ease diffusion of reactants and products during the HER, but it also promotes light harvesting and accelerates the transfer of photogenerated charge carriers from inner to the outer active sites results in enhancement photocatalytic HER activity. CN-B showed inferior HER activity due to its non-porous and solid agglomerate shape. This study indicated the significant effect of porous structure and P doping on enhancement the HER activity. In another study, Fang et al. realized the broader and stronger sub-bandgap adsorption by fabricating P-doped g-C_3_N_4_ nanoflakes (PCNNFs) using phytic acid and urea as P source and g-C_3_N_4_ precursor, respectively [95]. The ultrathin nanoflakes assembled in a porous network-like shaped enlarged surface area, with broad light adsorption of up to 800 nm, a reduced charge-to-surface migration path in in-vertical-plane and in-plane directions, and improved charge separation and transfer efficiency, which synergistically promoted the outstanding photocatalytic H_2_ production rate of 15,921 μmol h^−1^g^−1^ and a quantum efficiency of 6.74% at 420 nm. 

Thus, engineering the morphology of gCN nanostructures can benefit the separation and movement of photogenerated charge pairs, facilitate mass transfer, and offer more accessible active sites for catalytic reactions [6,28,100,101]. For instance, Zhu et al. proposed a template-free synthesis of mesoporous phosphorus-doped g-C_3_N_4_ nanoflowers (P-CN) with in-plane mesopores (3–18 nm) and open-up surface (Figure 5d) by co-condensation and thermolysis of a mixture with ME as g-C_3_N_4_ precursor and (hydroxyethylidene) diphosphonic acid as phosphonic source [76]. EDS mapping showed the presence of C, N, and P in P-CN; meanwhile, the XPS analysis demonstrated that P mainly replace C or N in g-C_3_N_4_ framework to form P-N or P-C bonds. Compared with pristine g-C_3_N_4_, mesoporous g-C_3_N_4_ and g-C_3_N_4_ nanosheets, the combination of novel morphology and P doping endows P-CN with excellent H_2_ evolution activity. This is seen in the higher H_2_ evolution rate of P-CN (104.1 μmol h^–1^) that is 9.29 times more than the bare g-C_3_N_4_ reference (11.2 μmol h^–1^), implying the effects of a porous flower-like shape and P-doping, which enhance the mobility of the charge carriers and HER photocurrent as further shown in (Figure 5e). Likewise, P-doped g-C_3_N_4_ micro-flowers composed of ultrathin nanosheets were obtained by using phosphoric acid as the P source and cyanuric acid–melamine complex as the supramolecular precursor [97]. The inimitable porous micro-flower, with P doping and high surface area, enhanced the utilization of visible light and promoted isolation separation and mobility in the photogenerated charges, which promoted the H_2_ rate by 24 times more than the bulk g-C_3_N_4_ flake-like structure. In addition to mesoporous P-doped g-C_3_N_4_ 3D nanoflower, one-dimensional P-doped hexagonal tubular carbon nitride (P-TCN) was reported in hydrothermal and pyrolysis processes using phosphorous acid and ME as raw materials [91]. As seen from the SEM image (Figure 5f) P-TCN had a hexagonal tube with the layered stack structure that comprised exposed rich pores of 40–60 nm along the tube wall as shown in the TEM image (inset in Figure 4f). According to the UV/Vis light absorption spectra and XPS valence band spectra, the electronic structure of P-TCN changed after P doping relative to bulk g-C_3_N_4_ (GCN); the corresponding band structure alignments are depicted in Figure 4g. Photoluminescence (PL) emission can be used to determine the trapping and transfer behavior of photoexcited charges. The PL intensity of P-TCN decreased significantly, indicating a lower recombination rate after P doping compared with GCN (Figure 5g) due to its hexagonal tube morphology, which provides a short path for the mobility of photogenerated charges and delays their recombination. Figure 5h presents the time course of HERs for GCN, TCN, and P-TCN, in which P-TCN displays the highest hydrogen evolution rate at 67 μmol h^−1^ because of P doping and its tubular structure. To add, no noticeable deterioration of H_2_ evolution rate was observed during four cycles of 20 h, suggesting the robust stability of P-TCN. During the linear sweep voltammetry (LSV) measurements (Figure 5i), the larger hydrogen evolution current density of P-TCN represented enhanced electron transfer compared to that of GCN. Additionally, P-TCN revealed an almost threefold enhancement in photocurrent response compared to GCN (Figure 5j). This implies the significant effect of porous hexagonal tube on boosting the specific surface area and active sites in addition to the effect of P doping on reducing band gap energy, enhancing the electric conductivity, and delaying the recombination of photogenerated electron–hole pairs, which lead to increment of the photocatalytic HER efficiency on P-TCN. A similar P-doped tubular g-C_3_N_4_ structure was also obtained via hydrothermal and thermal polymerization processes using ME and sodium pyrophosphate as starting precursors [92]. Afterwards, coral-like porous P-doped g-C_3_N_4_ tubes (PCNT) were developed by using dicyandiamide and phytic acid as starting materials [96]. The highest photocatalytic hydrogen evolution rate, 2020 μmol h^−1^g^−1^, was achieved on PCNT, which is about 4.7- and 22.4-fold compared to that of g-C_3_N_4_ tubes and pristine bulk g-C_3_N_4_, respectively. Additionally, Zhang et al. prepared P-doped macro/mesoporous g-C_3_N_4_ microrods (CNRs) by direct calcination of ethylene diphosphonic acid–ME complex fiber network [93]. Benefitting from unique morphology and electronic properties, the P-CNRs yield a 5.5 times higher hydrogen evolution rate than pristine g-C_3_N_4_. 

In most cases, the doped P atoms only substitute C or N atoms of g-C_3_N_4_ to form either a P-N or P-C bond [99,147]. Zhou et al. proposed a thermally induced copolymerization route for P-doped g-C_3_N_4_ by using hexachlorocyclotriphosphazene as a phosphorus source and guanidinium hydrochloride as g-C_3_N_4_ precursor, respectively [90]. X-ray photoelectron spectra (XPS) and NMR results revealed that the P atoms locate at the corner and bay carbon sites of g-C_3_N_4_ network to form P-N bond, and the lone electron from the P atom delocalizes to the Π-conjugated triazine ring. The P doping could modify the electronic structure, surface texture, and electric conductivity of g-C_3_N_4_, leading to improved photocatalytic activity. In another study, Sun and his coworkers realized dual-site doping by using ammonium hypophosphite as the P source, thus regulating the band structure of g-C_3_N_4_, accelerating the charge separation and transfer [98]. Various characterization results indicate that two forms of P-N coordination exist: one is the substitution of carbon atoms in the tris–triazine framework to form P-N bonds, the other is the formation of surface N-P-O bonds. Consequently, the dual-site P-doped g-C_3_N_4_ yields a 5.7-fold higher H_2_ evolution rate than the pristine catalyst.

#### 5.1.2. Sulfur Doping

**Figure 6:**
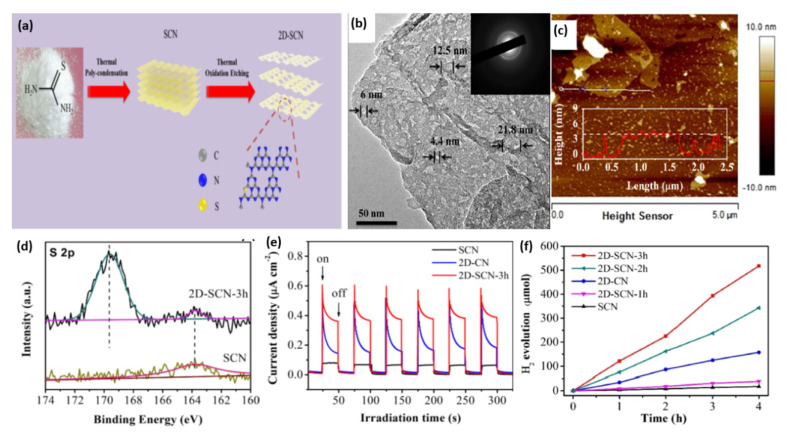
(**a**) Schematic illustration of the synthesis procedure of 2D-SCN. (**b**) TEM image and (**c**) AFM image of 2D-SCN-3h. (**d**) S 2p XPS spectra of SCN and 2D-SCN-3h. (**e**) Transient photocurrent–time curves, and (**f**) H_2_ production on 2D-SCN-3h, 2D-SCN-2h, 2D-SCN-1h, SCN, 2D-SCN, and 2D CN. Adapted with permission from Ref. [108]. 2020, American Chemical Society.

Sulfur doping has also been used to tune the bandgap and electronic structure of g-C_3_N_4_ for enhanced visible light absorption, improved charge separation and transfer, and consequently, photocatalytic performance [104,107,111,148]. For instance, Xu’s group fabricated S-doped mesoporous g-C_3_N_4_ (mpgCNS) by using thiourea as a precursor and SiO_2_ nanoparticles as a template [113]. As evidenced by XPS study, the S atoms tend to replace carbon sites to form an S-N bond. Compared with undoped mesoporous g-C_3_N_4_ (mpgCN), mpgCNS can further enhance light absorption in both UV and visible light regions. Moreover, the lowest PL intensity for mpgCNS implies the less recombination tendency of photogenerated electron–hole pairs. As expected, the H_2_ evolution rate of mpgCNS is 36% higher than that of mpgCN. Additionally, S-doped 2D graphitic carbon nitride nanosheets (2D-SCN) were prepared by the initial polycondensation of thiourea to in situ doped S into bulk g-C_3_N_4_ (denoted as SCN), followed by the thermal oxidation process, as illustrated in Figure 6a [108]. The bulk g-C_3_N_4_ (CN) and 2D g-C_3_N_4_ nanosheets (2D-CN) were synthesized using urea as a precursor. The thermal oxidation etching for different times 1, 2, and 3 h formed 2D-SCN-1h, 2D-SCN-2h, and 2D-SCN-3h, respectively. This led to a break in the hydrogen bond and reduced the thickness between the 2D layers of g-C_3_N_4_ nanosheets alongside the creation of a mesoporous structure. The TEM image of 2D-SCN-3h showed well-defined mesoporous nanosheets with multiple pores (4.4–21.8 nm) (Figure 6b) with an average thickness of 4.0 nm as shown by atomic force microscope (AFM) (Figure 6c). The surface area, porosity (i.e., pore volume and pore diameter), and H_2_ production increased significantly after oxidation etching. Thereby, the surface area (226.9 m^2^/g) and pore volume (0.81 cm^3^/g) of 2D-SCN-3h were superior to SCN by 13.66 and 6.23 times, respectively, due to their porous morphology. The XPS displays the presence of C, N, and S; meanwhile, the XPS spectrum of S 2p—in which the weak peak at 163.7 eV is related to the S-C bond by substituting lattice N (Figure 6d)—was not similar to the work mentioned above reported by Xu’s group. As seen from the transient photocurrent response for three samples in Figure 6e, 2D-SCN-3h possesses a higher current density than SCN due to its porous nanostructure and great surface area that increases active sites, which is beneficial for more highly efficient charge separation. By comparing the photocurrent between 2D-SCN-3h and 2D-CN, it is also proven that S doping can promote charge carrier separation and transfer. According to partial density of states computation, the S-C-N bonds formed by S doping are more effective than O-C-N bond. S doping into a porous morphology can also narrow the band gap and enhance light absorption, thus synergistically improving photocatalytic performance. This is shown in the higher H_2_ production rate (127.4 µmol/h) in 2D-SCN-3h than in 2D-SCN-2h (85.8 µmol/h), 2D-SCN-1h (9.7 µmol/h), 2D-CN (41.6 µmol/h), SCN (0.5 µmol/h), and CN (0.4 µmol/h) (Figure 6f). Zhou et al. further regulated the above polymerization process of thiourea by adding NH_4_Cl as a bubble template to obtain g-C_3_N_4_ with a porous network [106]. It is deduced that NH_4_Cl can decompose into NH_3_ and HCl during polymerization, which contributes to the forming of a porous structure and avoiding agglomeration. Apart from thiourea, trithiocyanuric acid also acts as a widely reported sulfur-containing precursor for S doping [105,110,112]. Luo and his co-workers proposed a one-step thermolysis of thiocyanuric acid approach to preparing ultrathin S-doped holey g-C_3_N_4_ nanosheets [110]. The optimum sample can deliver a superior H_2_ evolution rate of 6225.4 μmol g^−1^h^−1^ under visible light irradiation, almost 45-fold higher than pristine bulk g-C_3_N_4_. Additionally, an apparent quantum yield of 10% at 420 nm could be yielded. In addition to the nanosheets, nanoporous g-C_3_N_4_ microrods were prepared for photocatalytic HER by self-assembling melamine–trithiocyanuric acid supramolecule, followed by calcination [105]. 

#### 5.1.3. Boron Doping

**Figure 7:**
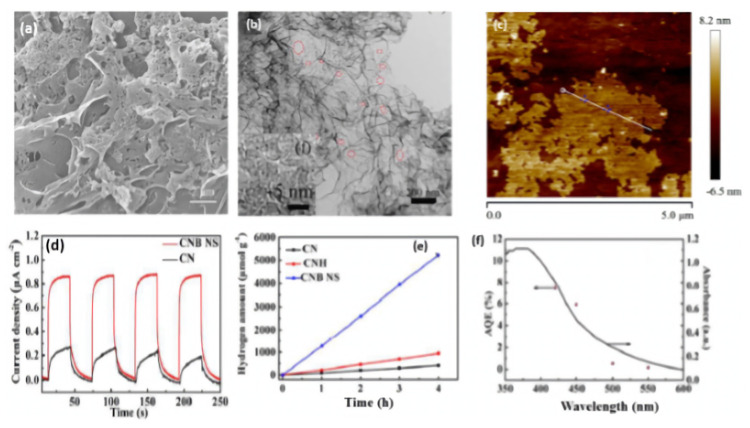
(**a**) SEM image, (**b**) TEM image, (**c**) AFM image of CNB NS. (**d**) Transient photocurrent–time curves, (**e**) H2 production amount on CNB NS, CNH, and CN nanocatalysts. (**f**) The wavelength-dependent AQE and absorption curve of CNBs. Reproduced with permission from Ref. [116]. 2018, Royal Society of Chemistry.

B-doped porous ultrathin g-C_3_N_4_ nanosheets (CNB NS) were prepared by the initial hydrothermal treatment of an aqueous solution of melamine and barbituric acid for 10 h at 180 °C, followed by annealing at 550 °C for 2 h [116]. In their synthesis, barbituric acid (BA) not only assemble with melamine to generate a porous structure but also replace the triazine ring of the heptazine unit in the g-C_3_N_4_ framework. As references, CNH was obtained via the hydrothermal and annealing methods but without barbituric acid and CN was synthesized by direct heating of melamine at 550 °C for 2 h. The SEM image of CNB NS showed the formation of porous nanosheets (Figure 7a) and the TEM image revealed that the nanosheets are ultrathin with multiple in-plane pores and curled edges (Figure 7b) with an average thickness of 3–4 nm (Figure 7c). This reflects the effect of annealing and hydrothermal treatment in presence of barbituric acid on the morphology as further seen by the BET surface area of CNB NS (55.07 m^2^/g) being 3.26 times greater than CN (16.88 m^2^/g). XPS analysis revealed the higher ratio of the C1s peak at 284.6 eV in CNB NS compared to in CN, owing to the incorporation of B-doping; however, the peak of the B-based dopant does not appear in the XPS survey and there is no other quantitative analysis to confirm presence of B. The UV-vis diffuse reflectance spectra (DRS) showed a red shift of the band edge absorption of CNB NS of 30 nm more than CN, implying that incorporating BA could decrease Π-deficiency and induce an asymmetric electron density. As reflected by the electronic band structure in Figure 7b, the conduction band of CNB NS shows a negative shift of 0.16 eV compared to that of CN, which implies the effect of a porous ultrathin curled nanosheet shape on reducing photoexcited electrons as seen in the lower band gap energy of CNBS (2.69 eV) than CN (2.78 eV), which is characteristic of the generation of photogenerated electron–hole pairs and delays their recombination. This is seen in the photocurrent–time curves, which indicate the quick and uniform photocurrent responses on all samples but with a superior activity on CNB NS (Figure 7d). The photocurrent responses were reversible under each dark/light circulation, which implied a higher photocurrent in light than under dark. Furthermore, CNB NS showed the highest hydrogen evolution rate at 1323.25 μmol h^−1^g^−1^, which is almost 13 times higher than that of CN (Figure 7e). This is due to its porous nanostructure with great surface area, which provides more exposed catalytic active sites along with the prevention of nanosheet aggregation during the HER as shown in the stability of CNB NS for five cycles without any apparent attenuation of the H_2_ evolution rate. Figure 7f displays the wavelength-dependent apparent quantum efficiency (AQE) of H_2_ evolution for CNB NS, and the AQE decreases with the increased wavelength due to the light-induced HER, implying enhanced visible light harvesting. In another study, B-doped carbon nitride nanotubes were fabricated by a simple hydrothermal treatment of boric acid and melamine, followed by calcination [117]. Although the band structure of carbon nitride was not changed after B doping, the fully exposed active sites and high-density charge carriers endowed them with a highly efficient and stable hydrogen evolution rate.

#### 5.1.4. Oxygen Doping

Oxygen-doped g-C_3_N_4_ was first prepared using a facile H_2_O_2_ hydrothermal approach [122]. As evidenced by the XPS result, O atoms could be incorporated into the matrix and substitute sp^2^-hybridized nitrogen atoms in g-C_3_N_4_ to form C-O and N-C-O species. Moreover, a negative shift in the conduction band minimum was observed after O doping, whereas the valence band maximum remained unchanged. As a result, the O doping could adjust the electronic and band structure, resulting in extended visible light response, enlarged surface areas, and suppressed charge carrier recombination. In another study, Huang et al. reported the synthesis of O-doped g-C_3_N_4_ with a porous network by condensation of supramolecular aggregates formed by melamine and H_2_O_2_ [123]. They found that the control over the O-dopant amount is significant, and excess dopants could serve as defect sites for electron–hole recombination. The optimal catalysts delivered 6.1 and 3.1 times higher HER activity than pristine and porous g-C_3_N_4_ and an apparent quantum efficiency of 7.8% at 420 nm. Guo and his coworkers synthesized holey-structured g-C_3_N_4_ with edge oxygen doping using a photo-Fenton reaction in the presence of Fe^3+^/Fe^2+^ and H_2_O_2_. Based on the various characterization technique, oxygen edge doping can remarkably broaden light harvesting capacity and improve charge separation efficiency. 

**Figure 8:**
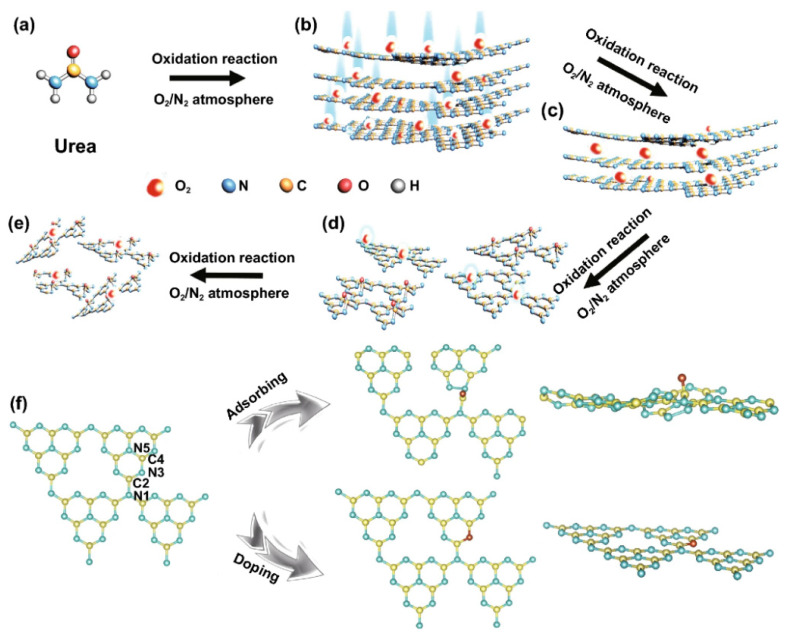
The formation process and mechanism of OCN photocatalysts. (**a**–**e**) synthesis of OCN photocatalysts. (**f**) DFT simulations. Reprinted with permission from Ref. [132]. 2021, Springer Nature Switzerland.

**Figure 9:**
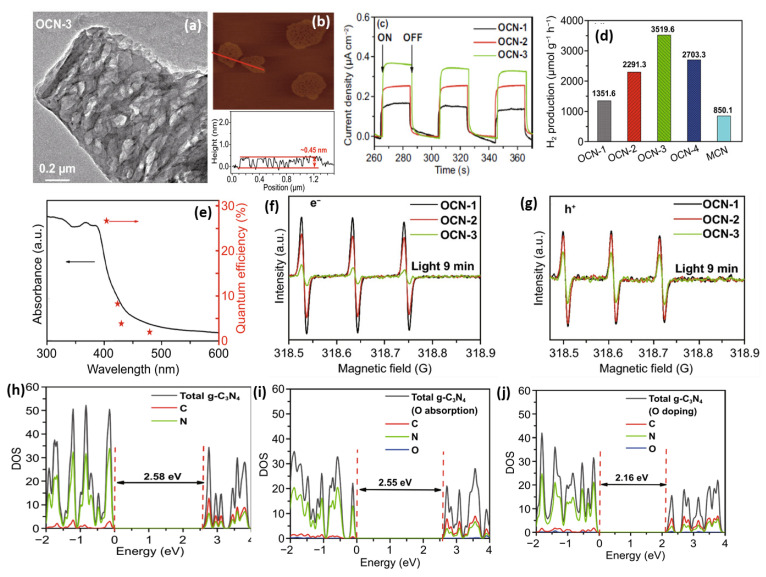
(**a**) TEM, (**b**) AFM image of OCN-3. (**c**) Transient photocurrent responses and (**d**) H_2_ production rate under optical filter (λ > 420 nm) of OCN-1, OCN-2, and OCN-3. (**e**) The QE–wavelength curve of OCN-3. (**f**) ESR of electron and (**g**) hole on OCN-1, OCN-2, and OCN-3. The density of state of (**h**) bare g-C_3_N_4_, (**i**) O-doped g-C_3_N_4_, and (**j**) O-CN. Adapted with permission from Ref. [132]. 2022, Elsevier Inc.

Apart from H_2_O_2_, Huang’s group introduced ammonium persulphate ((NH_4_)_2_S_2_O_8_) into the co-pyrolysis and polycondensation process of g-C_3_N_4_ [127]. Owing to its strong oxidative ability, (NH_4_)_2_S_2_O_8_ can inhibit polycondensation from creating nitrogen defects and contribute to the porous structure and oxygen dopants. Importantly, the pathway of electron transition was changed with respect to bulk g-C_3_N_4_, which significantly accelerated the charge separation. As expected, nearly sixfold higher photocatalytic activity than pristine g-C_3_N_4_ was observed for O-doped g-C_3_N_4_. Another typical example is that Wu et al. developed multiple thermal treatment methods to obtain hollow O-doped g-C_3_N_4_ nanosheets (OCN) under a N_2_/O_2_ atmosphere [132]. The formation mechanism was attributed to the adsorption of the O atom in g-C_3_N_4_ under the initial thermal treatment of urea in the N_2_/O_2_ atmosphere to form OCN-1, which oxidized and allowed integration of more O-doping after being retreated thermally under the N_2_/O_2_ to form OCN-2 and then oxidized again to form OCN-3 and OCN-4 (Figure 8). The same concept could be feasible for allowing the doping of other atoms with O via simple mixing with sources for heteroatoms (i.e., thiourea and boron trichloride). Meanwhile, bulk g-C_3_N_4_ (MCN) was synthesized under identical conditions with OCN-1 except for using melamine under an argon atmosphere. The TEM image (Figure 9a) and AFM image (Figure 9b) of OCN-3 showed the formation of porous monolayered nanosheets with uniform distribution of abundant pores (25 nm size) and the thickness of the sheet was ~0.45 nm. The surface area, porosity, and O-content increased with increasing the thermal treatment times, but the OCN-3 possessed the highest surface area of (148.50 m^2^/g^1^) compared to OCN-2 (102.84 m^2^/g^1^) and OCN-1 (97.88 m^2^/g^1^); the high surface area of OCN-3 is beneficial for promoting the HER. XPS analysis revealed that the O content increased from 0.84 at% in OCN-1 to 1.62% in OCN-3 and 2.07% in OCN-4. The photocurrent response revealed that OCN-3 had a higher photocurrent density (0.35 μA/cm^2^) than that of OCN-1 (0.14 μA/cm^2^) and OCN-2 (0.26 μA/cm^2^) (Figure 9c) in addition to durability for five cycles over 20 h with only 10.4% loss, but the current density was very low in all samples and the difference between the photocurrent and electrochemical current was inferior. Under visible light irradiation for 5 h, the hydrogen evolution activity of OCN-3 is the highest among these catalysts (Figure 9d). The quantum yield of OCN-3 decreases with increased wavelengths, and it can reach 26.96% at 400 nm and 4.28% at 420 nm (Figure 9e) due to the ability of OCN-3 to induce the generation of electron–hole pairs along with delaying their recombination. This is shown in the electron spin resonance spectroscopy (ESR) analysis with TEMPO, which showed that OCN-3 had a weak signal that is an inaction for a large number of photo-exited electron–hole pairs relative to OCN-1 and OCN-2, implying the significant effect of porous monolayer sheet morphology and higher O-doping (Figure 9f,g). That the signals for electron–hole pairing under light irradiation reduced more slowly over time than its counterparts is evidence of the efficient isolation of photo-exited radicals. Density functional theory was applied to clarify the enhanced photocatalytic mechanism of O atoms. Based on the density of states, the band gap of pure g-C_3_N_4_, O-adsorbed g-C_3_N_4_, and O-doped g-C_3_N_4_ is about 2.58, 2.55, 2.16 eV, respectively (Figure 9h–j). Thus, it can be concluded that introducing oxygen atoms can narrow the band gap for harvesting more visible light, promoting the generation and isolation of electron–hole pairs, while porous nanosheets with high surface area provide more accessible active catalytic sites and accelerate the charge mobility and diffusion of reactants or products during the HER. 

#### 5.1.5. Carbon Doping

Li et al. proposed a facile microwave-assisted heating approach to yield C-incorporated g-C_3_N_4_ by copolymerizing Π-electron-rich barbituric acid with melamine [135]. Theory calculations showed that carbon incorporation provides more available Π-electrons, shortens the bandgap, and in addition, microwave irradiation could improve the crystallinity of g-C_3_N_4_, benefiting the rapid charge transfer. The H_2_ production amount of C-incorporated g-C_3_N_4_ is almost 20 times higher than that of g-C_3_N_4_ synthesized by the conventional heating method. Chen and coworkers realized the simultaneous introduction of carbon atoms and nitrogen vacancies in g-C_3_N_4_ by a combined hydrothermal–conjugate–copolymerization strategy [136]. Three important features were mentioned: (1) the porous structure could provide more available active sites for reaction species adsorption, (2) the defects-induced midgap could broaden the visible light absorption, (3) the gradient C-doping could promote charge carrier transfer, enabling excellent photocatalytic activity for C-doped g-C_3_N_4_.

**Figure 10:**
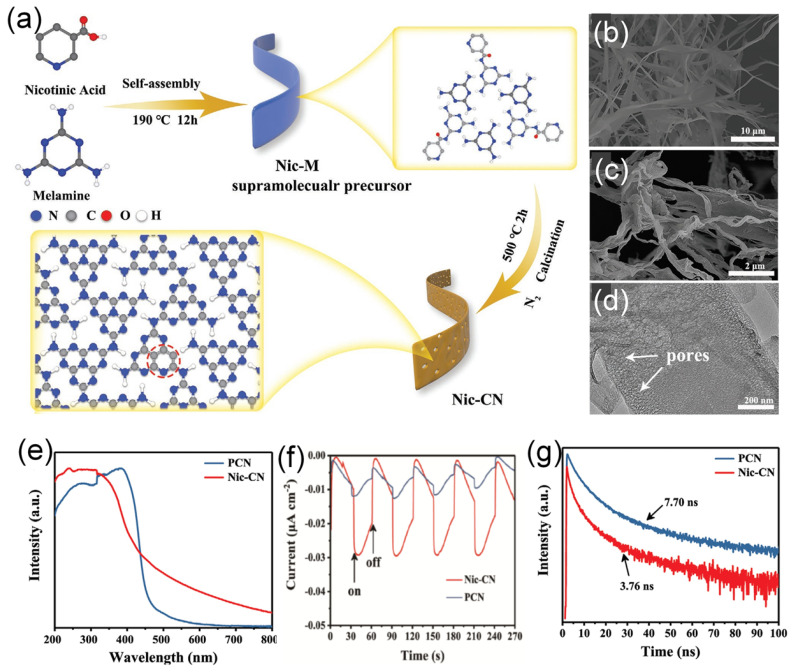
(**a**) Schematic illustration of the synthesis process of Nic-CN. SEM image of (**b**) Nic-M precursor and (**c**) Nic-CN. (**d**) TEM image of Nic-CN. (**e**) UV-vis DRS of PCN and Nic-CN, (**f**) transient photocurrent responses of PCN and Nic-CN, and (**g**) transient state PL spectra of PCN and Nic-CN. Adapted with permission from Ref. [138]. 2021, John Wiley & Sons, Inc.

Given the unique structural advantage, 1D porous strip-like carbon nitride was prepared by calcining supramolecular precursor (Nic-M) formed by molecular self-assembly of melamine and nicotinic acid (Figure 10a) [138]. The SEM image (Figure 10b) of Nic-CN exhibits a strip nanostructure morphology with about 1 μm width; meanwhile, Nic-M preserved strip-like morphology well (Figure 10c). The TEM image (Figure 10d) shows the thinner strip features of Nic-CN with pores distributed on the surface, which can benefit the photocatalytic reaction. The XPS and elemental analysis confirmed the C-doping as shown by the higher C content in Nic-CN (47.06%) than PCN (45.14%). The UV-vis DRS (Figure 10e) showed the adsorption peak at 200–400 nm with a band edge extended to nearly 460 nm in PCN and 460–700 nm in Nic-CN, due to the 1D porous strip-like morphology and modification of pyridine groups. This led to broadened optical absorption of Nic-CN by a threshold of up to 700 nm more than PCN and enhanced the separation efficiency of photogenerated electron–hole pairs. Moreover, the extended conjugation resulted in a downshift of the conduction band edge and, thus, a narrower bandgap of Nic-CN 2.65 eV than PCN (2.71 eV). Compared with PCN, the lower PL peak intensity of Nic-CN suggests inhibitive recombination and enhanced separation of carriers (Figure 10f). The transient state PL spectra show the shorter lifetime of Nic-CN, which implies the faster transfer of photogenerated charge pairs and efficient separation of charge carriers. Thereby, the photocurrent responses of Nic-CN were higher and quicker than of PCN, implying superior transport (Figure 10g). The photocurrent density of Nic-CN was higher than that of dark current by nearly (1.5 times). Unexpectedly, a nearly 18 times higher hydrogen evolution rate (126.2 μmol h^−1^), was achieved for Nic-CN than that for PCN, due to its porous 1D strip-like shape with high surface area, more exposed and accessible active sites, as well as better optical absorption during the HER. This is shown in the higher surface area of Nic-CN (40.95 m^2^/g) being four times more than PCN. In another report, carbon doping carbon nitride with a hollow tubular structure was fabricated for enhanced photocatalytic performance via a simple hydrothermal calcination method by using melamine and sodium alginate as precursors [139].

#### 5.1.6. Nitrogen Doping

The co-thermal condensation of a precursor with a nitrogen-rich additive is a common way of preparing N-doped g-C_3_N_4_ [140,142]. For instance, Fang et al. reported nitrogen self-doped graphitic carbon nitride for the first time by using melamine pretreated with hydrazine hydrate as the starting material [140]. The N/C mass ratio of 1.68 for the typical sample determined by elemental analysis was higher than that of 1.60 for g-C_3_N_4_ synthesized with melamine, indicating successful nitrogen incorporation. The as-prepared catalysts yielded nearly 14 times higher H2 evolution activity than pristine g-C3N4. Similarly, Shi’s group prepared N-doped porous g-C3N4 nanosheets by calcining urea and N-N dimethylformamide (DMF) [124]. DMF not only affords a N source for N doping but also releases dimethylamine to generate pores in the g-C_3_N_4_ framework. Nitrogen-rich carbon nitride nanotubes (CNNTs) were fabricated via the thermal polycondensation of supramolecular intermediates [141]. On one hand, their hollow tubular structure can provide more active sites for light adsorption and more refraction pathways for charge separation. On the other hand, N atoms with larger electronegativity doping could attract more electrons for photogenerated charge carrier transportation.

#### 5.1.7. Halogen Doping

It is well accepted that halogen doping of g-C_3_N_4_ can narrow the band gap, expedite the transfer of photogenerated electron–hole pairs, and enlarge specific surface areas, thereby improving its photocatalytic properties [143,144,145,146]. Wen’s group proposed a universal route to synthesizing halogen (e.g., Cl, F)-doped g-C_3_N_4_ for enhanced photocatalytic hydrogen evolution performance [144]. They found that the F or Cl atom with a higher electronegativity tends to replace the N atom and bond to the C atom. Among them, ultrathin Cl-doped g-C_3_N_4_ nanostrips possess the strongest light absorption, a narrow band gap, larger specific surface areas, and faster charge separation and migration, thus delivering an excellent H_2_ evolution rate of 5976 μmol h^−1^g^−1^. Gao et al. studied carbon nitride’s iodine surface modification and doping by heating the self-assembly precursors formed by urea and ammonium iodide [145]. As an electron donor, iodine doping could increase the electron density in carbon nitride networks and tune their band structure. Moreover, the surface iodine could bond with positively charged holes to hinder the recombination of photogenerated charge pairs.

### 5.2. Binary Heteroatom-Doped Porous Carbon Nitride

Binary heteroatom doping could integrate the advantage of a single dopant, thus synergistically benefiting the photocatalytic activity [149,150,151,152,153,154,155,156,157,158,159]. The detailed structure and performance comparison of binary heteroatom-co-doped gCN-based catalysts is listed in Table 7. Additionally, the effect of morphology, preparation methods, and light source on the H_2_ production rate and stability on binary doped porous gCNs is summarized in Table 7.

Cui et al. prepared B- and F-co-doped g-C_3_N_4_ via thermal polymerization of dicyandiamide, urea, and ionic tetrafluoroborate liquids, followed by post-annealing treatment [150]. The B atoms mainly located in the inside skeleton of g-C_3_N_4_, whereas F atoms existed in the surface layer. It was found that inner B doping contributes to enhancing visible light absorption and generating uniform porous structures during post-calcination treatment. Afterward, the same group reported the synthesis of porous C-I-co-doped carbon nitride with a similar method except for using an iodized ionic liquid as a precursor [153]. In another study, boron/oxygen-co-doped g-C_3_N_4_ nanomesh was obtained by a two-step doping and etching approach [151]. The as-prepared sample possessed a two-dimensional porous structure with a specific surface area of 160.58 m^2^ g^−1^.

**Figure 11:**
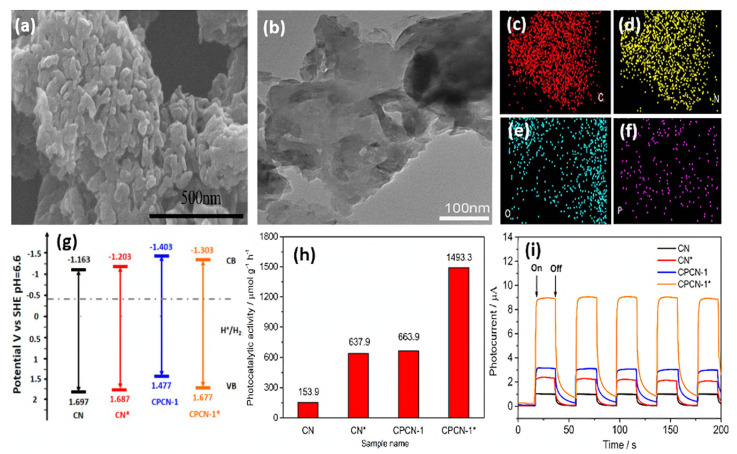
(**a**) SEM image, (**b**) TEM image, and (**c**–**f**) element mapping analysis of CPCN-1*. (**g**) Electronic band gap structure, (**h**) H_2_ production rate, and (**i**) transient photocurrent responses of CN, CN*, CPCN-1, and CPCN-1*. Adapted with permission from Ref. [154]. 2017, American Chemical Society.

Moreover, doped B and O atoms can regulate the band gap. Owing to the synergistic effect of the nanomesh-like structure and B- and O-co-doping, the light absorption ability and charge separation efficiency of g-C_3_N_4_ were enhanced. As a result, they displayed an excellent H_2_ evolution rate of 9751 μmol h^−1^g^−1^ under visible light illumination, which is almost 28 times higher than bulk g-C_3_N_4_. More importantly, the H_2_ evolution activity kept over 20 h, revealing its robust stability. Dai’s group proposed a facile approach to prepare C- and P-co-doped g-C_3_N_4_ (CPCN-1*) by the first self-assembly of melamine with phytic acid (denoted as CPCN-1), followed by hydrothermal treatment [154]. For comparison, pristine g-C_3_N_4_ (CN) was synthesized under identical conditions with CPCN-1 other than phytic acid, and CN* was obtained after hydrothermal treatment of CN. The SEM image of CPCN-1*, showed the formation of porous granular morphology (Figure 11a) with a rough surface and curled edges (Figure 11b); meanwhile, element mapping analysis revealed the presence of C, N, O, and P (Figure 11c–f). The surface area CPCN-1* (141.1 m^2^/g) was significantly higher than that of CN (24.9 m^2^/g) CN* (75.7 m^2^/g) and CPCN-1 (44.8 m^2^/g), due to the porous morphology and co-doping effect. Apparently, C and P doping could not change the band gap significantly but decreased the conduction band position of CPCN-1 and CPCN-1* in comparison with that of CN and CN*, implying much-enhanced photo-reducibility after co-doping (Figure 11g). Thus, CPCN-1 and CPCN-1* allowed for efficient improvement in the transfer of photogenerated electron–hole pairs over undoped CN and CN* because the dopants acted as trapping sites to restrain the combination of charge carriers. XPS analysis showed that the content of P dopants was found to be 0.29% in CPCN-1 compared to 0.14% in CPCN-1*. The H_2_ production rate on CPCN-1* (1493.3 μmol h^−1^g^−1^) was 2.24, 2.34, and 9.7 times that of CPCN-1 (663.7 μmol h^−1^g^−1^), CN* (637.6 μmol h^−1^g^−1^), and CN (153.9 μmol h^−1^g^−1^), respectively (Figure 11h). This implies porous morphology with a higher surface area and co-doping lead to high optical adsorption, efficient charge transport, and great photoinduced reducibility. Moreover, CPCN-1* showed high stability over four cycles for 13 h. The photocurrent responses over five on–off cycles revealed the obvious enhancement in the current density under light than under dark with reversible behavior and the photocurrent intensity remained stable during the five cycles, but CPCN-1* was the most active (nearly eight times CN) (Figure 11i). Beyond the above observation, the integration of binary heteroatom-doping and a heterojunction structure into g-C_3_N_4_ was also realized for enhanced photocatalytic performance [155,160].

### 5.3. Ternary Heteroatom-Doped Porous Carbon Nitride

**Figure 12:**
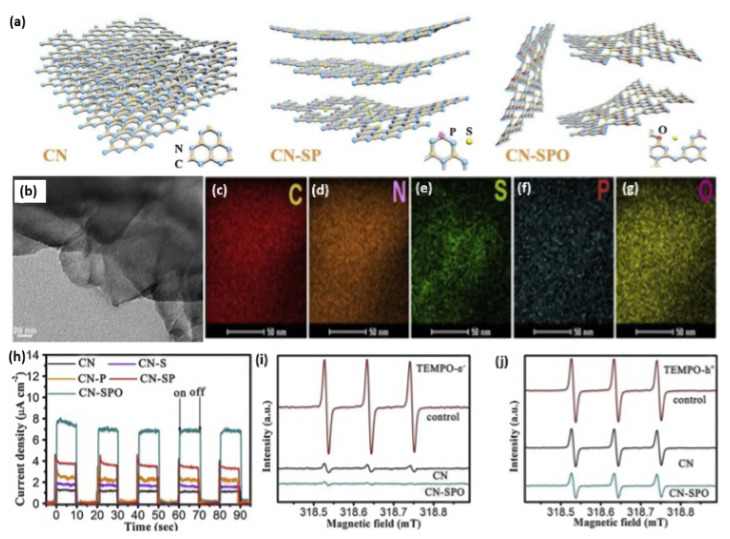
(**a**) Schematic illustration of exfoliation process of CN, CNSP, and CN-SPO. (**b**) TEM image and (**c**–**g**) EDX mapping of CN-SPO. (**h**) Transient photocurrent responses of CN-SPO and its counterparts. (**i**) ESR of electron and (**j**) hole of CN-SPO and CN. Adapted with permission from Ref. [161]. 2019, Elsevier Inc.

It has been proven that ternary heteroatom doping can show higher photocatalytic activity and unusual physiochemical properties with respect to binary and single heteroatom doping. For instance, Liu and coworkers realized the simultaneous doping and exfoliation of g-C_3_N_4_ by fabricating S-, P-, and O-co-doped ultrathin nanosheets via a facile annealing method [161]. As displayed in Figure 12a, S- and P-co-doped g-C_3_N_4_ (denoted as CN-SP) was first obtained by thermal condensation of the mixed precursors of melamine, thiourea, and diammonium phosphate. Subsequently, the CN-SP was annealed in the air for O doping; meanwhile, it could be exfoliated into ultrathin nanosheets (denoted as CN-SPO). The TEM image of CN-SPO ultrathin (~3 nm) exfoliated nanosheets morphology with sharp edges flat surface (Figure 12b), while CN and CN-SP had thicker tightly stacked sheets. The EDX analysis of CN-SPO clearly warranted the coherent distribution of C, N, S, P, and O, but the exact content is not mentioned in the manuscript (Figure 12c–g). Moreover, the XPS analysis of CN-SPO displayed the presence of C, N, S, P, and O. Experimental and theoretical results demonstrated that the S atoms occurred on the interstitial sites, whereas P and O atoms substituted C and N atoms, respectively. S, P, and O doping can probably create a more favorable charge transfer channel and boost charge migration. Accordingly, CN-SPO displayed the highest photocurrent response (8 μA/cm^2^), compared to CN-SP and CN, implying the significant effect of S/P/O doping on the optimal separation rate of photogenerated carriers under visible light irradiation (Figure 12h). This is seen in the ESR analyses, which displayed a lower TEMPO-e^-^ and TEMPO-h^+^ TEMPO with three peaks (with ratio of 1/1/1) but with a less intensity on CN-SPO than CN under light than under dark (Figure 12i,j). This implied the superior reactivity of photoinduced charge carriers on CN-SPO. In addition, the narrow band gap and negatively shifting conduction band edge caused by heteroatom doping could broaden visible light absorption and enhance electron reducibility. As a result, an optimal H_2_ evolution rate of (2480 μmol g^−1^ h^−1^) was observed for CN-SPO, which was superior to CN (465 μmol g^−1^ h^−1^) by five times. This is due to the tri-dopant, which promoted the photoexcited electrons transfer through the (O-P-C-N_2_-S-N_2_ or N_2_-S-N_2_-C_1_-P-O) chain among the two adjacent heptazine units, which eased the isolation of the photogenerated carriers. 

**Figure 13:**
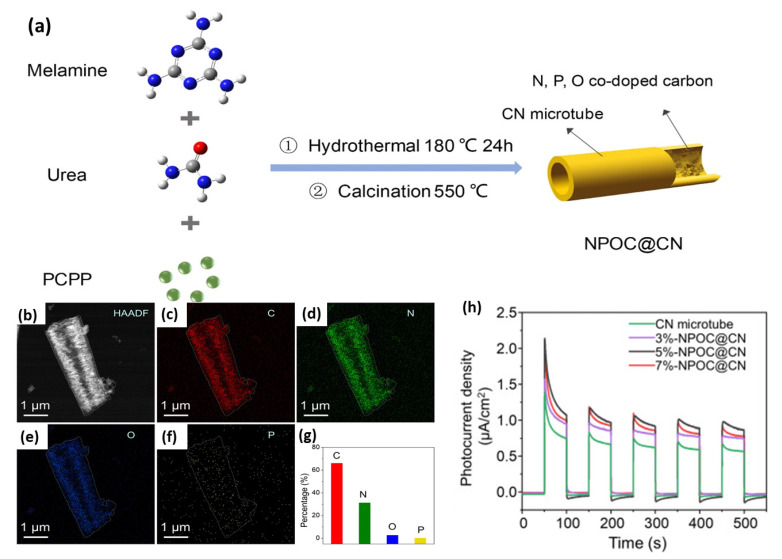
(**a**) The formation process, (**b**) HAADF-STEM, (**c**–**g**) EDX mapping of 5%-NPOC@CN. (**h**) transient photocurrent responses on 5%-NPOC@CN relative to its counterparts. Adapted with permission from Ref. [162]. 2022, Elsevier Inc.

N-, P-, and O-co-doped carbon (NPOC)-filled CN microtubes (NPOC@CN) were synthesized via the initial mixing of urea and melamine with the poly(cyclotriphosphazene-*co*-phloroglucinol) (PCPP) microspheres and then hydrothermal and annealing too (Figure 13a). A 3, 5, and 7% amount of PCPP was used for preparation of 3%-NPOC@CN, 5%-NPOC@CN, and 7%-NPOC@CN, correspondingly. The high angle annular dark field (HAADF) image of 5%-NPOC@CN revealed a porous microtube structure with multiple pores (mesopore, micropore, and macropores) with the highest specific surface area of (68.75 m^2^/g^1^) (Figure 13b). The EDX mapping displayed that the atomic contents of C, N, O, and Pare 66.05, 31.20, 2.59, and 0.15%, respectively (Figure 13c). The bandgap energy of 5%-NPOC@CN (2.18 eV) was lower than that of 7%-NPOC@CN (2.31 eV), 3%-NPOC@CN (2.43 eV), and CN (2.63 eV), implying better light harvesting and isolation efficiency of photogenerated carriers due to the tri-dopant effect. Furthermore, the H_2_ production rate on 5%-NPOC@CN (1149.71 μmol g^−1^ h^−1^) was 1.32, 1.58, 2.06, and 112.60 times greater than that of 7%-NPOC@CN (869.13 μmol g^−1^ h^−1^), 3%-NPOC@CN (724.43 μmol g^−1^ h^−1^), CN (556.89 μmol g^−1^ h^−1^), and bulk CN (10.21 μmol g^−1^ h^−1^), respectively. This is also shown in the quicker response and higher photocurrent density of 5%-NPOC@CN than its counterpart (Figure 13d). All samples showed a noticed difference between photocurrent and electrochemical current. Thereby, tri-doping could pave the way for creation of novel CN-based photocatalysts for HER, but it is rarely reported and should be explored. 

## 6. Conclusions and Prospective

This review highlighted the synthesis of non-metal-doped porous gCNs for the photocatalytic HER. This includes the utilization of H_2_ as a fuel and its storage in addition to the fundamentals related to water electrolysis and the photocatalytic HER process (i.e., mechanism, measurements, and calculation) and non-metal dopant configuration into gCNs. Meanwhile, the effect of non-metal dopants (i.e., mono, binary, and ternary heteroatom (i.e., P, O, S, N, and B)-doped porous gCN nanostructures on the enhancement of photocatalytic H_2_ production were also discussed as a function of photocatalyst shape, composition, bandgap, and electrolyte type or concentration on HER activity and durability. 

Mono-non-metal-doped porous gCNs are studied more than binary-doped, while ternary-doped gCN is rarely reported; however, mono-doped gCNs are the most active and promising for the HER. B-CNNT obtained via the hydrothermal and calcination of method in the presence of boric acid revealed a H_2_ rate of (22,100 μmol h^−1^g^−1^) [117] relative to CNNTs formed via the supermolecule self-assembly method that showed a H_2_ rate of (18,060 μmol h^−1^g^−1^) [141], and PCNNFs fragmented nanoflakes obtained via doping and annealing of biomass (15,921 μmol h^−1^g^−1^) [95]. Binary BO-C_3_N_4_ formed via a two-step doping and etching method is among the most promising photocatalysts for HERs, with a H_2_ evolution rate of (9751 μmol h^−1^g^−1^) [151] in addition to high durability for 20 h. Moreover, p-CN-BF obtained via in situ co-doping using [Emin]BF_4_ and calcination in air yielded a H_2_ rate of (7020 h^−1^g^−1^) [150]; however, both materials needed Pt as a co-catalyst. Ternary CN-SPO showed a H_2_ evolution rate of (2480 μmol g^−1^ h^−1^) [161] due to the presence of S atoms on the interstitial sites and substitution of C with P and N atoms with O atoms in gCN, resulting in lower bandgap and higher light absorption efficiency. The supramolecular self-assembly of multiple nitrogen-enriched carbon precursors such as melamine with cyanuric acid [105] and urea with ammonium iodide [145] are effective in the formation of porous gCN nanostructures with high surface area. This is in addition to facilitate in situ doping with non-metal atoms during the self-assembly process (i.e., S-doping in case of self-assembly of thiourea, O doping using cyanuric acid, and B doping using boric acid). Although there has been substantial progress made in the synthesis of porous, doped gCN photocatalysts for HERs, they remained impractical for large-scale applications and various perspectives and barriers are still unaddressed:

- Previous porous, doped gCNs in the form of 2D nanosheets and other porous nanostructures are rarely reported or not yet reported. Porous multidimensional doped gCN (i.e., nanoflower, nanodendrite, yolk–shell, and nanocage) and one-dimensional (i.e., nanowires, nanotubes, nanorods, and nanotubes) morphologies are imminent with their impressive characteristics (i.e., high electrical conductivity, great surface area, abundant defects, massive active/accessible active sites, stabilization of metal/non-metal atoms, and maximized atomic utilization) [163]. These merits can endow the HER activity and the durability of doped porous gCNs. Such porous nanostructures could be synthesized using multiple nitrogen-rich carbon precursors containing non-metal elements (i.e., melamine, thiourea, cyanuric acid, and cyanimide) and changing the preparation conditions (i.e., annealing environment, templates, and solvent type) [28,163]. Meanwhile, the reported g-doped porous gCNs are powder, which cannot be used directly in electrolysis and require several steps to be used as a cathode. This could be realized via the in situ fabrication of gCNs on solid carbon-cloth sheets or metal hydroxide/oxide substrate that could be used as a cathode for the HER.
- Particular attention should be paid to developing facile, one-step, and eco-friendly methods to fabricate g-C_3_N_4_ with various morphologies. Recently, our group developed a simple, template-free, one-pot approach for the fabrication of porous one-dimensional gCN nanostructures (i.e., wires, fibers, tubes, and rods) in situ, doped with various metals (i.e., Au, Pd, Pt, Cu, and their combinations) with high surface area and outstanding catalytic properties for CO oxidation [3,4,5,164]. The same tactics can be extended to prepare other gCN structures with various single-atom metals, dopants, and nanoparticles for CO_2_ reduction. Single-atom-impeded g-C_3_N_4_ for CO_2_ reduction is not studied enough. g-C_3_N_4_ comprises a triazine or heptazine skeleton that can accommodate various single-metal atoms to maximize atom utilization; minimize attrition; reduce deactivation; and enhance CO_2_ activity, selectivity, and durability.
- Both experimental and theoretical calculations/simulations (i.e., DFT and artificial intelligence) could be coupled to understand the effect of non-metal dopants on the physicochemical properties of porous gCN nanostructures and their catalytic/photocatalytic activities and mechanisms.
- The relatively high overpotential, low current densities, and inferior long-term stability are critical barriers in gCNs for HERs, which cannot meet practical requirements (i.e., current density up to several ambers and durability for several weeks or months). This could be solved using noble metal dopants in the formation of heterojunction structures with porous metal oxynitride [165,166], multimetallic nanocrystals [12,167,168], MXenes [2,169,170,171,172], MOF [10,173,174], graphene [175], and graphdiyne [176] to augment solar light harvesting and charge carrier separation during the HER.
- The safety of H_2_ storage tanks should be considered because in the case of unexpected accidents, the H_2_ tank becomes a bomb. Defeating these barriers requires using high-pressure vessels made of fiber-based composites that can afford a high pressure of up to 700 bar and subsequently can improve cold or cryo-compressed hydrogen storage along with boosting H_2_ density and using novel, durable, low-cost materials for H_2_ adsorption [18,46,47,48]. Moreover, using novel adsorbents for H_2_ storage is safer than tanks, but needs more efforts to decrease operation conditions (i.e., pressure) and enhance storage capacity.

## Figures and Tables

**Table 1:** Summary of the main reviews related to porous heteroatom-doped porous carbon nitride-based nanostructures for photocatalytic HER compared in the present review.

Title	Focus	Ref.
Non-metal-Doped Porous Carbon Nitride Nanostructures for Photocatalytic Green Hydrogen Production	Rational design of heteroatoms (i.e., B, N, S, P, F, and O) doped porous carbon nitride for the photocatalytic HER. The effect of mono, binary, and ternary dopants on photocatalytic HER, and their fundamentals and mechanisms are discussed. H_2_ energy and storage in addition to HER fundamentals and calculation are also discussed. The current challenges and possible solutions for the synthesis of active gCN photocatalysts for green HERs are also emphasized.	This work
Photocatalytic hydrogen evolution based on carbon nitride and organic semiconductors	Organic (i.e., carbon nitride, linear polymers, conjugated porous polymers, and small molecules) for a photocatalytic HER.	[33]
Solvent-Assisted Synthesis of Supramolecular-Assembled Graphitic Carbon Nitride for Visible Light Induced Hydrogen Evolution—A Review	Solvent (i.e., water, DMSO, and water–chloroform) assisted the supramolecular-assembled carbon nitride via hydrogen bonding and hydrogen–halogen interaction. This is in addition to tunable characteristics/properties of photocatalytic HERs.	[34]
A latest overview on photocatalytic application of g-C_3_N_4_ based nanostructured materials for hydrogen production	g-C_3_N_4_ nanosheets supported metals (i.e., transition metals, non-metals, noble, and semiconductor), and carbon materials (i.e., graphene and carbon nanotubes, and carbon dots) for photocatalytic HER.	[35]
Preparation, Physicochemical Properties, and Functional Characteristics of Carbon Nitride: a Review	Emphasizing various approaches for preparation, and functionalization of porous carbon nitride and their properties that could enhance the photocatalysis, catalysis, and adsorption applications.	[41]
gC_3_N_4_ Derived Materials for Photocatalytic Hydrogen Production: A Mini Review on Design Strategies	Highlighting the recent advance in doped g-C_3_N_4_ with metals/non-metals (i.e., Ag, Ni, Mo, F, B, and S) and formation of heterojunction with semiconductors (i.e., TiO_2_, ZnO, MCoS_2_), metal-organic framework, graphdiyne for photocatalytic HERs, and their mechanisms.	[38]
Latest progress in g-C_3_N_4_ based heterojunctions for hydrogen production via photocatalytic water splitting: a mini review	The fabrication of g-C_3_N_4_-based heterojunctions (i.e., type-II, Z-scheme, S-scheme and Schottky) with transition metal oxide/sulfide (i.e., Co_2_P, FeOx, CuS, Cu_2_O, Ni) noble-metals (i.e., Ag, Au, Pt, and Pd), non-metals (i.e., B, F, S, and W), semiconductors (ZnO, ZrO_2_, and Mo_2_S), and carbon materials (graphene, carbon, nanotubes, and carbon dots) for photocatalytic HERs.	[36]
2D Graphitic Carbon Nitride for Energy Conversion and Storage	The preparation (i.e., thermal oxidation etching, chemical exfoliation, ultrasonication-assisted liquid phase exfoliation, chemical vapor deposition) of energy production (i.e., photo-/electrocatalytic HER, CO_2_ reduction, and oxygen evolution/reduction) and energy storage (i.e., alkali-metal ion, lithium-metal, lithium-sulfur batteries, metal-air batteries, and supercapacitors).	[42]
Recent advances on porous materials for synergetic adsorption and photocatalysis	Focus on the fabrication of porous g-C_3_N_4_, metal oxides/sulfides (i.e., ZnS, SnS_2_, BiS_3_), metal-organic frameworks (i.e., ZIF, MIL, and PCN) for photocatalytic HERs, and photocatalytic adsorbents (TiO_2_-actvated carbon, activated carbon-MoS_2_, and biochar-TiO_2_).	[37]
Co-Doped, Tri-Doped, and Rare-Earth-Doped g-C_3_N_4_ for Photocatalytic Applications: State-of-the-Art	Unravelling the effects of co-doping, tri-doping, and rare-earth-doping of g-C_3_N_4_ with non-metals (i.e., P, F, N, I, S, Cl) and metals (Ag, Fe, Co, and Pt) on photocatalytic water splitting and dye degradation.	[39]
Recent advances in g-C_3_N_4_-based photocatalysts incorporated by MXenes and their derivatives	The fabrication of g-C_3_N_4_/2D MXenes (I.e., Ti_3_C_2_T_x_, Nb_2_CT_x_) and their derivatives for environmental and energy applications (i.e., photocatalytic HER, generation, CO_2_ conversion, pollutant degradation, N_2_ fixation, and H_2_O_2_ production).	[43]

**Table 2:** HER steps in an aqueous solution of acidic and alkaline electrolytes.

Name	Equation	Equation No.
HER in an aqueous solution of acidic electrolyte		
Volmer reaction step	gCNs-M* + H_3_O^+^ + e^−^ → gCNs-M*H_ads_ +H_2_O	(4)
Heyrovsky reaction step	gCNs-M*H_ads_ + H_3_O^+^ + e^−^ → gCNs-M* + H_2_ +H_2_O	(5)
Tafel reaction step	gCNs-M*H_ads_ + gCNs-M*H_ads_ → 2gCNs-M* + H_2_	(6)
HER in an aqueous solution of alkaline electrolyte		
Volmer reaction step	gCNs-M* + H_2_O + e^−^ → gCNs-M*H_ads_ + OH^−^	(7)
Heyrovsky reaction step	gCNs-M*H_ads_ + H_2_O + e^−^ → gCNs-M* + H_2_ + OH	(8)
Tafel reaction step	gCNs-M*H_ads_ + gCNs-M*H_ads_ → 2gCNs-M* + H_2_	(9)

**Table 3:** HER measurements and calculations.

Name	Equation	Equation No.
Quantum efficiency (QE)	QE (%) =$\;\frac{\mathrm{number}\;\mathrm{of}\;\mathrm{reacted}\;\mathrm{electrons}\;}{\mathrm{number}\;\mathrm{of}\;\mathrm{incident}\;\mathrm{photons}}\times 100$	(10)
Current density (J)	J = I/A	(11)
Over potential (η)	η = E − Eo	(12)
Turnover frequency (TOF)	TOF = JA/2Fm	(13)
Reduction current density vs. scan rate	Slope = 2nFAΓ0/4RT	(14)
The turnover frequency (TOF)	TOF = J × NA/(F × n × Γ)	(15)
Energy efficiency (Eefficiency)	Eefficiency = [Eeq/Eeq + η] × EFaradic	(16)
Quantum yield	QY= [2.nx.NA.h.c/till.I.A.λ]	(17)
Electrochemical active surface area (ECSA)	ECSA = CDL/Cs	(18)
Double-layer capacitance (Cdl)	*Cdl* = (Δ*j*)/2*dVb*	(19)
Incident photon to current conversion efficiency	IPCE (%) = [(1240 × J)/(λ × Io)] × 100	(20)

**Table 4:** Comparison of the advantages and disadvantages of mono-heteroatom-doped porous gCNs.

Doping	Advantages	Disadvantages
N	Generates abundant defects and active sites Increases electronic conductivity Promotes ion adsorption Earth-abundant and inexpensive Modulates the Fermi level, bandgap, and localized electronic state Eases the generation of electron–hole pairs and delays their recombination Enhances light absorption	Not durable at elevated temperatures Does not enlarge interlay distance Uncontrolled doping sites and concentration Cumbersome process High operating temperature
S	Expands interlayer distance Induces reduction reaction Promotes ion adsorption/diffusion Modulates the Fermi level, bandgap, and localized electronic state Facilitates the generation of electron–hole pairs and delays their recombination Enhances light absorption	Leads to structural deformation Uncontrolled doping sites and concentration High operating temperature Slow preparation process
P	Enlarges interlayer distance Enhances ion adsorption/diffusion Upsurges geometric distortion Alters the Fermi level, bandgap, and localized electronic state Induces creation of electron–hole pairs and prevents their quick recombination Enhances light absorption	Causes large structural distortion Uncontrolled doping sites and concentration High operating temperature Slow preparation process
B	Generates massive in-plane defect Enhances ion adsorption/diffusion Modulates the Fermi level, bandgap, localized electronic state, and spin density Induces creation of electron–hole pairs and prevents their quick recombination Enhances light absorption	Difficult to prepare Forms high-energy trap Uncontrolled doping sites and concentration
F	Enlarges interlayer distance Enhances ion adsorption/diffusion Enhances electronic conductivity Suppresses the John–Teller effect Modulates the Fermi level, bandgap, and localized electronic state	Causes large structural deformation upon cycling Excess doping causes rapid capacity disappearance Hazardous precursors Uncontrolled doping sites and concentration

**Table 5:** Comparison between the preparation methods of doped gCN nanostructures based on data from Refs. [60,80,81,82,83,84,85,86,87,88].

Methods	Precursors	Doping	Advantages and Disadvantages
Thermal annealing	Boric trioxide Boron trichloride Boron trioxide Ammonia Diammonium hydrogen Urea Hydrogen sulfide Diaminodiphenyl sulfone Dibenzyl sulfide Sulfur powder Hexachlorocyclotriphosphazene Phosphoric acid Diammonium phosphate Hexafluorophosphate Ammonium fluoride Ammonium chloride Ammonium bromide	B B B N N N S S S S P P P P F Cl Br	Simple, one-pot, feasible for various precursors (i.e., gases, liquids, and solids), tunable doping. Limitations: high operation temperature and energy consumption
Physical vapor deposition (PVD) or chemical vapor deposition (CVD)	Boric acid Phenylboronic acid Ammonia Iodine Pyrimidine	B B N I N	Allows simultaneous growth of doped gCNs with controllable doping Limitations: complex process, energy consumption, requires special laboratory equipment, and generates waste gases Limitations: high cost, inferior yield, and feasible for low ranges of precursors
Ball milling	Ammonia sulfur powder Ammonium fluoride Ammonium chloride Ammonium bromide	N S F Cl Br	Low-cost, facile, and scalable process Limitations: doping only at edges Limitations: uncontrolled doping process
Bottom-up synthesis	Boron tribromide Lithium nitride Pentachloropyridine Thiourea	B N N S	Highly productive, solution-based, need mild conditions Limitations: inevitable high oxygen content and uncontrollable doping
Wet chemical method	Hydrazine Ammonium thiocyanate Hydrogen fluoride Hydrogen iodide Ammonium chloride Ammonium bromide Boric acid	N S&N F I Cl Br B	Inexpensive, low energy consumption, solution-based, productive, easy process, and feasible for wide ranges of precursors Limitations: low-doping content and uncontrollable doping
Plasma	N_2_ Cl_2_ Hydrogen sulfide	N Cl D	Quick process and inferior power consumption Limitations: low yield and feasible for specific precursors
Arc-discharge	NH_3_ Pyrrole Boron trioxide	N N B	Productive and quick process Limitation: high energy consumption (i.e., voltage and current) Limitation: inferior and uncontrollable doping content

**Table 6:** Comparison of photocatalytic performance of mono heteroatom doped porous carbon nitride toward HER.

Photocatalysts	Doping Element	Morphology	Synthetic Method	Co-Catalyst	Light Source	H_2_ Evolution Rate (μmol h^−1^g^−1^)	Apparent Quantum Efficiency	Durability	Ref
P-CN	P	Mesoporous nanostructured flowers	Template-free co-condensation method	3 wt% Pt	300 W Xeon arc lamp	2082		No obvious attenuation of H_2_ evolution rate after illumination of 16 h	[76]
PCN-S	P	Porous nanosheets	Thermal polycondensation of melamine-2-aminoethylphosphonic acid complex, followed by thermal exfoliation	1 wt% Pt	300 W Xe arc lamp with a UV-cutoff filter (>400 nm)	1596	3.56% at 420 nm		[89]
P10-550	P	Layered platelet-like morphology	Thermally induced copolymerization route using hexachlorocyclotriphosphazene as P source and guanidinium hydrochloride as g-C_3_N_4_ precursor. (10 wt%P, calcination temperature = 550 °C)	3 wt% Pt	300 W xenon lamp with a 420 nm cutoff filter	506		The hydrogen amount is still comparable to that of first cycle after five cycles	[90]
P-TCN	P	Hexagonal tubes with micro-nanostructure	Pyrolysis of the melamine–cyanuric acid supramolecular precursor formed by phosphorous acid-assisted hydrothermal method	1 wt% Pt	300 W Xeon arc lamp with bandpass filter (365, 420, 450, 520, and 600 nm)	670	5.68% at 420 nm	No noticeable deterioration after irradiation for 20 h	[91]
CN-SP	P	Tubular g-C_3_N_4_ with surface carbon defects	Thermal polymerization of a supramolecular precursor formed under pyrophosphate–assisted hydrothermal process	1 wt% Pt	300 W Xe arc lamp with a ≥420 nm cutoff filter	570			[92]
P-CNRs	P	Macro/mesoporous g-C_3_N_4_ micro-rods	Direct calcination of reflux-treated ethylene diphosphonic acid–melamine complex fiber network	3 wt% Pt	300 W Xe arc lamp with a ≥420 nm cutoff filter	4960		No obvious decay after irradiation for 20 h	[93]
P0.01	P, Na	Porous multi-layer nanosheets	Polymerization of the mixed precursors of melamine and sodium tripolyphosphate	1 wt% Pt	350 W Xe arc lamp	3820		No decrease in H_2_ production rate after irradiation for 12 h	[94]
PCNNFs	P	Fragmented nanoflakes	First P-doping via using phytic acid biomass as P source and urea as C_3_N_4_ precursor, followed by posttreatment	3 wt% Pt	300 W Xe arc lamp with a >420 nm cutoff filter	15,921	6.74% at 420 nm; 0.24% at 600 nm	No obvious decay in photocatalytic H_2_ production under irradiation for 50 h	[95]
PCNT	P	Hierarchical coral-like porous tubes	Pyrolysis and freeze-drying using dicyandiamide as carbon nitride source and phytic acid as P source	3 wt% Pt	300 W Xe arc lamp with a ≥420 nm cutoff filter	2020	4.32% at 420 nm; 3.58% at 450 nm; 1.28% at 500 nm	H_2_ production rate kept almost same after 10 h reaction	[96]
PCN1.5	P	Flower-like structure consisting of multitudinous nanosheets	Template-free and thermal copolymerization route using phosphoric acid as P source and cyanuric acid–melamine complex as supramolecular precursor	3 wt% Pt	300 W Xe lamp with a 400 nm UV-light cutoff filter	5128		Only about 7.3% attenuation was observed after visible light illumination of 16 h	[97]
PCN-50	P	Platelet-like surface	Polymerization of urea and NH_4_H_2_PO_2_ at 570 °C for 3 h	1 wt% Pt	300 W Xe lamp with a 400 nm UV-light cutoff filter	~9167		Photocatalytic performance was maintained through 20 h of cycling experiments	[98]
PCN(1.6)	P	Nearly transparent nanosheets agglomerate	The calcination of polymeric carbon nitride formed by urea condensation and amorphous phosphorus	3 wt% Pt	300 W Xe arc lamp as simulated sunlight (>300 nm) or with a 420 nm cutoff filter	8707 and 5720 under the simulated solar light and visible light			[99]
P-CNTS	P	Tubular structure with a large number of pores in the walls	Pre-hydrothermal and calcination under a nitrogen atmosphere	1 wt% Pt	300 W Xe lamp with a 420 nm UV-light cutoff filter	2749.3		The amount of produced hydrogen slightly decreased after three cycles of tests	[100]
L-PCN-1.0	P	Louver-like nanowire arrays	Supramolecular self-assembly of melamine–cyanuric acid	1 wt% Pt	300 W Xe arc lamp with a ≥420 nm cutoff filter	1872.9	6.93% at 420 ± 15 nm	The hydrogen production has no noticeable deactivation over four cycles	[101]
PCN-HMS	P	Hierarchical mesoporous microspheres	Supramolecular chemistry-mediated one-pot strategy	1 wt% Pt	300 W Xe lamp with an ultraviolet cut-off filter (λ ≥ 420 nm)	1820			[102]
PO-CN	P	Porous ultrathin nanosheets	Two-step thermal treatment	3 wt% Pt	300 W Xe lamp with a 420 nm cutoff filter	997.7			[103]
NiSCN	S, Ni	Nanosheets	High-temperature thermal polymerization of urea and benzyl disulfide	5 wt% Ni	300 W Xe arc lamp with a cutoff filter (λ > 420 nm)	2021.3	2.51% at 420 nm	The H_2_ production rate decreases a little after four cycles for 20 h	[104]
MTCN-6	S	Rectangular rods	Self-assembly of melamine with tri-thiocyanuric acid, followed by calcination	1 wt% Pt	300 W Xe arc lamp with a cutoff filter (λ > 420 nm)	1511.2	3.9% at 420 nm	No obvious decrease in H_2_ generation rate over five cycles	[105]
PCNS-2	S	Ultrathin nanosheets with porous networks	The polymerization of thiourea and NH_4_Cl at 550 °C for 3 h		300 W Xe arc lamp with a cutoff filter (λ > 420 nm)	~367		No obvious decrease of H_2_ evolution rate within four cycles	[106]
CN-0.20%Dx-25	S,K	Needle-like nanorods	Condensation of thiourea and dithiooxamide followed by post-treatment in molten salt	Pt	300 W Xe arc lamp with a cutoff filter (λ > 420 nm)	1962.10		Obvious decrease in the photocatalytic H_2_ evolution performance due to K leaching	[107]
2D-SCN	S	Nanosheets	Polycondensation of thiourea, followed by thermal oxidative treatment	1 wt% Pt	140 W Xe lamp	8493	8.35% at 420 nm	The hydrogen evolution activity was maintained after 36 h of continuous irradiation	[108]
S/g-C_3+x_N_4+y_	S, cyano group	Porous leaf with irregular shape	Treating pristine g-C_3_N_4_ nanosheets under acetonitrile and hydrogen sulfide atmosphere	3 wt% Pt	300 W Xe lamp with a UV light filter (λ > 420 nm)	1901	33.5% and 13.1% at 405 and 420 nm	The amount of produced hydrogen was decreased in first three cycles, but returned to the previous high level after the re-addition of TEOA sacrificial agent	[109]
S-CN(0.1)	S	Holey nanosheets	One-step thermolysis of thiocyanuric acid	3 wt% Pt	300 W Xe lamp with a UV light filter (λ > 420 nm)	6225.4	10% at 420 ± 10 nm	The photocatalytic HER stabilizes at ca. 6200 μmol h^−1^g^−1^ under five cycles of reuse	[110]
PCNS	S	Layered structure	One-step auxiliary thermal polycondensation of melamine and ammonium persulfate	2 wt% Pt	300 W Xenon lamp with a 420 nm UV-cutoff	58,680		No obvious decrease of H_2_ production after three cycles	[111]
SCN1.0	S	Peony-like morphology	Thermal condensation of cyanuric acid–melamine–trithiocyanuric acid complex under N_2_ atmosphere	3 wt% Pt	300 W Xe lamp	11,354	13.69% at 420 nm	Only about 3.2% attenuation of photocatalytic hydrogen production after four cycles	[112]
mpgCNS	S	Mesoporous nanosheet	Pyrolysis of thiourea using SiO_2_ nanoparticles as the hard template	3 wt% Pt	300 W Xe lamp with a 420 nm cutoff filter	1360	5.3% at 420 nm	10% activity drop over the photoreaction for 72 h with evacuation at every 12 h	[113]
CN-MT	S	Nanoporous microrods	Thermal condensation of melamine-trithiocyanuric acid supramolecular cocrystal under N_2_ atmosphere	1 wt% Pt	500 W Xe lamp with a 400 nm filter	5000		No loss of catalytic activity after the catalytic H_2_ evolution for 60 h	[114]
SCN-HMS	S	Mesoporous microspheres	Supramolecular chemistry-mediated one-pot strategy	1 wt% Pt	300W Xe lamp with an ultraviolet cutoff filter (λ ≥ 420 nm)	2230	3.8% at 420 nm	No obvious decrease was observed for the H_2_ evolution rate even after four cycles	[102]
B-SSCN	B	Microsphere	One-step solvothermal method by using cyanuric chloride and cyanuric acid as precursors and ammonia borane as B source	3 wt% Pt	300 W Xenon lamp with a UV cutoff filter (λ > 420 nm)	910	~1.15% at 420 nm	The H_2_ evolution rate was well preserved after four test cycles over 4 days and no structure change after reaction	[115]
CNB NS	B	Porous ultrathin nanosheet	Reforming and thermal condensation of barbituric acid and melamine	3 wt% Pt	300 W Xe lamp with a UV cutoff filter (λ > 400 nm)	1323.25	7.45% at 420 nm	The amount of H_2_ production increase steadily with extended the reaction time and no significant deactivation is observed after five cycles	[116]
B-CNNT	B	Ordered nanotubes	Hydrothermal and calcination of melamine and boric acid	Pt	300 W Xenon lamp with a UV cutoff filter (λ > 420 nm)	22,100	7.33% at 420 nm	The H_2_ production increases steadily with time and retain stability	[117]
B, CsCN-Ns	B, Cs	Porous and wrinkled nanosheets	Recrystallization of melamine in water in the presence of boric acid and CsCl followed by calcination and thermal etching	3 wt% Pt	Xenon lamp with a cutoff filter (λ ≥ 420 nm) and IR filter	1120		The H_2_ production rate was stable in five successive cycles	[118]
B/g-C_3_N_4_	B	Nanosheets	The pyrolysis of urea and 1-ethyl-3-methylimidazolium tetrafluoroborate	0	350 W Xenon lamp with a UV cutoff filter (λ > 365 nm)	901		The photocatalytic activity remains unchanged after three reaction cycles	[119]
PNCN-BNa-3	B, Na	Porous nanosheets	Controlling the heating rate and thermal posttreatment using melamine nitrate as precursor, sodium borohydride as B source and Na source	1.2 wt% Pt	10 W white LED lamp (λ > 420 nm) with the color temperature of 6500 K	5971.51	9.39% at 430 nm	Even after five cycles of photocatalytic test, the hydrogen generation activity is not significantly reduced	[120]
D-TCN_450_	B	Hollow tube	Self-supramolecular reaction and NaBH_4_ thermal reduction approach	3 wt% Pt	300 W Xe lamp with a 420 nm cutoff filter	789.2			[121]
O-doped g-C_3_N_4_	O	Irregular porous structure with hierarchical edges	Hydrothermal treatment of g-C_3_N_4_ with H_2_O_2_ at 140 °C for 10 h	1.2 wt% Pt	300W Xe arc lamp with a UV-cutoff filter (λ < 420 nm)	375		The H_2_ evolution remains stable in the recycling three runs	[122]
MCN	O	Porous network composed by nanosheets	Condensation of supramolecular aggregates formed by H_2_O_2_-treated melamine	3 wt% Pt	300 W Xenon lamp with a 420 nm filter	1204	7.8% at 420 nm	A stable HER rate within 25 h	[123]
HS-g-C_3_N_4_-O	O	Holey thin sheets	Using photo-Fenton reaction in the presence of Fe^3+^/Fe^2+^ and H_2_O_2_	5 wt% Pt	300W Xe lamp with 420 nm filter	6752		The H_2_ evolution rate is quite stable under continuous irradiation of 26 h	[124]
P-CNO	O	Porous nanosheet with highly ordered architecture	Heating the hydrothermally treated dicyandiamide at 550 °C for 2 h	1 wt% Pt	300 W Xeon lamp with a UV cutoff filter (λ > 400 nm)	1748.6	7.2% at 420 nm	The hydrogen production performance shows no trend of deactivation even after 15 h	[125]
GCN-4	O	Three-dimensional porous nanosheets	Water-based homogeneous supramolecular assembly	3 wt% Pt	300 W Xeon-lamp equipped with a 420 nm-cutoff filter	1968	10.3% at 420 nm	A stable HER rate after six cycling trips	[126]
p-CN2	O	Loose and porous layers	A simple co-pyrolysis of dicyandiamide and ammonium persulphate	3 wt% Pt	300 W Xeon lamp with a UV cutoff filter (λ > 420 nm)	395.96	0.79% at 420 nm	The photocatalytic H_2_ production activity is well retained after four successive cycles while the phase structures are not changed	[127]
POCN	O	Nanosheet	Thermal polymerization reaction of melamine and ethanol	1 wt% Pt	300 W Xe lamp equipped with a 420 nm cutoff filter	1286	12.06% at 420 nm	The HER is no apparent attenuation after four cycles	[128]
CN3	O	Numerous macropores and mesopores with an assembling flake	Pyrolyzing H_2_SO_4_ and HNO_3_ modified melamine precursors	3 wt% Pt	300 W Xe arc lamp with an AM 1.5 optical filter	3700	20.88% at 420 nm	No significant decline of H_2_ production is observed after five runs within 5 h	[129]
W,O/g-C_3_N_4_	W/O	Hollow tubular structure	One-step polycondensation of ammonium metatungstate hydrate and melamine	1 wt% Pt	300 W Xe lamp with a cutoff filter (λ > 400 nm)	403.57			[130]
U/AC_0.5_	O	Loose and rich bread-like porous structure	Thermal polymerization of urea and foaming agent azodicarbonamide	3 wt% Pt	300 W Xe lamp equipped with a 420 nm cutoff filter	4470	13.0% at 400 nm	No significant decrease in photocatalytic performance after five cycles	[131]
OCN-3	O	Hollow and monolayered nanosheet	Multiple thermal treatments under the N_2_/O_2_ atmosphere	3 wt% Pt	300 W Xenon lamp with an optical filter (λ > 420 nm)	3519.6	26.96% at 400 nm	Only 10.4% activity loss after 20 h	[132]
FCN15	O	Rod	Calcinating supramolecular precursors prepared from acid (or alkali) and melamine	3 wt% Pt	300 W Xenon lamp	12,766	9.4% at 420 nm	The FT-IR and Raman spectra did not change significantly after 16 h cycle test	[133]
OCNT	O	Ultralong hollow chain-ball	Facile supramolecular self-assembly route	2 wt% Pt	300 W Xe lamp equipped with a 420 and 510 nm cutoff filter	5470	9.4% at 420 nm and 2.1% at 510 nm	The hydrogen production rate did not attenuate after six consecutive photocatalytic reactions	[134]
m-CN-0.067	C	Nanosheet	Copolymerizing barbituric acid with melamine via microwave-assisted heating	0.5 wt% Pt	300 W Xe lamp equipped with a 420 nm cutoff filter	2500		They offer slightly reduced H_2_ generation during the course of 15 h visible light irradiation	[135]
C-rich g-C_3_N_4_	C	Nanosheet	A hydrothermal–conjugate–copolymerization strategy	0	300 W Xe lamp equipped with a 420 nm cutoff filter	125.1	6.8% at 420 nm	No noticeable deterioration of stability activity is observed after three cycles test	[136]
CDCN-20	C	Thinner nanosheet enriched with many small holes	Co-polymerization of dicyandiamide with acrylamide	3 wt% Pt	300 W Xe lamp with a 420 nm filter	1266.8	10.14% at 420 nm	No apparent decrease in photocatalytic activity is observed after four cycling test	[137]
Nic-CN	C	1D thin, porous strip-like structure	Calcination of strip-like supramolecular precursor formed by organic molecular self-assembly of melamine and nicotinic acid	1 wt% Pt	300 W Xe lamp (λ > 420 nm)	6310	6.8% at 420 nm	No obvious attenuation in hydrogen evolution after 5 times of cycling	[138]
CN-40	C	Hollow tubular structure	Hydrothermal calcination method using melamine and sodium alginate as precursors	3 wt% Pt	300 W Xe lamp with a 420 or 400 nm cutoff filter	1210.3	3.16% at 420 nm	More than 56% of original performance can be remained after four runs of reaction	[139]
C_3_N_4+X_	N	Nanosheet	Co-thermal condensation of precursor with nitrogen-rich additive	3 wt% Pt	300 W Xeon lamp with a 400 nm cutoff filter	553.5		The amount of produced hydrogen increased linearly with the consecutive irradiation time	[140]
CNNTs	N	Hollow nanotube	Supermolecule self-assembly method	3 wt% Pt	300 W Xe lamp (λ > 420 nm)	18,060	12.55% at 420 nm	The photocatalytic hydrogen evolution kept stable over four cycles	[141]
CNU-DMF	N	Porous nanosheets	One-step thermal copolymerization of urea and N,N-dimethylformamide	3 wt% Pt	300 W Xe lamp (λ > 400 nm)	5268	11.4% at 420 nm	No obvious decrease after four cycles of reaction within 16 h	[142]
Cl-pdg-CN-M-3	Cl	Accumulation of thin sheets	Pyrolysis of the mixture of melamine and NH_4_Cl	3 wt% Pt	300 W Xe lamp with a 400 nm cutoff filter	833		The H_2_ produced increased steadily with irradiation time lengthened in each run without noticeable deactivation	[143]
Cl-p-C_3_N_4_	Cl	Ultrathin nanostrips	Calcination of melamine and tetrachloroterephthalonitrile in an inert atmosphere	1 wt% Pt	300 W Xenon lamp with a 420 nm cutoff filter	5976	8.91% at 420 nm	The H_2_ production rate has no apparent inactivation after four cycles	[144]
CNI	I	Loose and porous structure	Calcination of self-assembly precursors prepared from urea and ammonium iodide	1 wt% Pt	300 W Xe lamp equipped with a 420 nm cutoff filter	3800	3.3% at 420 nm	Excellent cycle stability in photocatalytic hydrogen production	[145]
CNU-Br_0.1_	Br	Layered platelet-like and curl-like thin nanosheet	A facile co-condensation strategy by using urea and ammonia bromine as starting materials	3 wt% Pt	300 W Xe-lamp equipped with an appropriate long pass cutoff filter	240		No activity decrease was seen after four consecutive cycles’ reaction	[146]

**Table 7:** Comparison of photocatalytic performance of binary heteroatom-co-doped porous carbon nitrides toward HER.

Photocatalysts	Dopants	Morphology	Synthetic Method	Co-Catalyst	Light Source	H_2_ Evolution Rate (μmol h^−1^g^−1^)	Apparent Quantum Efficiency	Durability	Refs
r-CN-B/F	B, F	Dense aggregated microstructures comprising irregular nanosheets	Post-thermal treatment of B/F co-doped carbon nitride obtained from direct condensation using ionic liquid as dopant	3 wt% Pt	Visible light irradiation (λ > 400 nm)	6870		High H_2_ evolution remains in the consecutive four runs	[149]
p-CN-BF	B, F	Small particles composed of porous nanosheets	In situ B and F co-doping using [Emin]BF_4_ as dopants followed by post-calcination in air	3 wt% Pt	300 W Xe arc lamp	7020		Only slight decrease observed in H_2_ evolution after several cycles	[150]
BO-C_3_N_4_	B,O	Porous nanomesh	Two-step doping and etching	3 wt% Pt	300 W Xe arc lamp equipped with a cutoff filter (λ ≥ 420 nm)	9751	8.1% at 420 nm	There was no obvious deactivation over 20 h	[151]
CNBS	B,S	Nanosheets composed of nanoholes and rupture	Co-pyrolysis of boric acid, thiourea and melamine in the muffle furnace	1 wt% Pt	150 W Xenon lamp with a 420 nm cutoff filter	2660		No decrease in hydrogen production rate during long-time photocatalytic measurement up to five runs	[152]
CNIN_0.2_	C,I	Unconsolidated porous accumulation	In situ co-doping with iodized ionic liquid followed by post-thermal treatment in air	3 wt% Pt	300 W xenon-lamp with appropriate cutoff filter	3364		Almost negligible deactivation could be observed after four consecutive cycles	[153]
CPCN-1*	P,C	Granular morphology	Self-assembly melamine with phytic acid, followed by hydrothermal treatment	1 wt% Pt	300W Xe lamp	1493.3	2.14% at 420 nm	The H_2_ generation rate recovered to initial value over three cycles	[154]
PSCN	P,S	Layered structure	One-step high-temperature polymerization	3 wt% Pt	300 W Xenon lamp with an UV-cutoff filter > 400 nm	1969		The hydrogen evolution was not apparently attenuated following three cycles’ running	[155]
PACN	P,O	Spiral nanotube	Phytic acid-assisted supramolecular self-assembly method	Pt	5 W LED lights	6437.65		PACN exhibited excellent recycling stability in four runs and maintained 95.20% of the primitive value of hydrogen evolution after four runs	[156]
CNB	C,O	Wrinkled nanosheets	Hydrothermal method using dicyandiamide, cyanuric acid and cyanobenzene as precursors	3 wt% Pt	300 W Xe-lamp with a 420 nm cutoff filter	2595.4	16.6% at 420 nm	The photocatalytic activity decreased slightly after five cycles and no noticeable change of microstructure could be found	[157]
POCN-10	P,O	Nanosheet	One-step thermal copolymerization of melamine and ammonium polyphosphate	5 wt% Pt	300 W Xenon lamp equipped with a 400 nm cutoff filter	1588		No apparent decline of H_2_ evolution activity by cycling three times in 12 h	[158]
SPCN0.1	S,P	Porous microtube	Using melamine and ammonium dihydrogen phosphate as precursors	3 wt% Pt	300 W Xe lamp equipped with optical cutoff filter (λ > 420 nm)	4200.3	10.3% at 420 nm	The performance did not significantly decrease after 12 h of experiment	[159]

## Data Availability

The data presented in this study are available on request from the corresponding authors.
